# Genomic analysis of red-tide water bloomed with *Heterosigma akashiwo* in Geoje

**DOI:** 10.7717/peerj.4854

**Published:** 2018-05-29

**Authors:** Hye-Eun Kang, Tae-Ho Yoon, Sunyoung Yoon, Hak Jun Kim, Hyun Park, Chang-Keun Kang, Hyun-Woo Kim

**Affiliations:** 1 Department of Marine Biology, Pukyong National University, Busan, Republic of Korea; 2 Interdisciplinary program of Biomedical, Mechanical and Electrical Engineering, Pukyong National University, Busan, Republic of Korea; 3 Department of Chemistry, Pukyong National University, Busan, Republic of Korea; 4 Korea Polar Research Institute, Korea Ocean Research and Development Institute, Busan, Republic of Korea; 5 School of Environmental Science and Engineering, Gwangju Institute of Science and Technology, Gwangju, Republic of Korea

**Keywords:** Red-tide, HABs, Algal bloom, PCR, Coastal water, Next generation sequencing, Microorganism

## Abstract

Microbial community structures of harmful algal bloom (HAB) caused by *Heterosigma akashiwo* in Geoje were analyzed using the MiSeq platform. To analyze phytoplankton communities without cross-reactivity with predominant bacteria, a new phytoplankton-specific 23S universal primer set was designed by modifying two previously used ones. The new universal primer set turned out to be a useful tool for the analysis of the phytoplankton community; it showed a high specificity for phytoplankton without cross-reactivity to bacterial sequences as well as the wide taxon coverage presenting from prokaryotic cyanobacteria to eukaryotic algae. Next Generation Sequencing (NGS) data generated by two universal primer sets (16S and 23S) provided useful information about the *H. akashiwo* bloom*.* According to the 23S universal primer set, proportions of *H. akashiwo* increased by more than 200-fold as the bloom occurred and its numbers were high enough to detect in control sites. Its operational taxonomic units (OTUs) were detected in the bloom sites at low proportions suggesting that the 16S universal primer set may not be as effective for monitoring harmful algal blooming (HAB) as the 23S universal primer set. In addition, several abundant OTUs in Chlorophyta were not presented by the 16S universal primer set in this study. However, the 16S primer set was useful for detecting decreases in Foraminifera as HAB occurred suggesting that genomic analyses using two universal primer sets would provide more reliable data for understanding microbial community changes by various environmental or ecological events, including HAB. Genomic analyses using two universal primer sets was also useful for determining a correlation between microbial components as HAB occurred. *Heterosigma akashiwo* was positively correlated with other bloom species, including *Karenia mikimotoi, Teleaulax amphioxeia,* and bacteria in Verrucomicrobia.

## Introduction

Red tide is a common name for the algal bloom of a few species of phytoplankton in coastal waters, which takes on a red or brown color depending on the type of algae (Glibert et al., 2005). These are also referred to as harmful algal blooms (HABs), which emphasizes their harmfulness (Smayda, 1997). The noxiousness of HABs is not limited to the health of people and marine ecosystems in the affected regions, it also adversely affects local and regional economies. In Korea, direct losses due to HAB from 2001 to 2012 amounted to about 52 million US dollars (Lee et al., 2014a). To develop a way to reduce the adverse effects of HABs, understanding their underlying mechanisms and early forecasting are crucial. Although many factors are known to contribute to HABs, including nutrient loadings and pollution (Anderson, Glibert & Burkholder, 2002; Moore et al., 2008; Sellner, Doucette & Kirkpatrick, 2003; Smayda, 1989), food web alterations (Anderson, 2009), introduced species (Hallegraeff, 1992), water flow modifications (Lee et al., 2014b; Sellner, Doucette & Kirkpatrick, 2003), and climate change (Peperzak, 2003; Wells et al., 2015), we still do not fully understand the complex interactions between factors which can create such an explosive growth in algal colonies.

Regular phytoplankton surveys are now being performed to monitor blooms in many countries, including Korea. Traditional phytoplankton surveys are conducted by optical observations in which each species and its numbers are identified based on their distinct morphological characteristics and counted. However, species-specific morphological characteristics are often indistinguishable, and it requires a lot of time and effort by well-trained experts. These have been the major obstacles preventing long-term or large-scale surveys. Recently, molecular techniques are being utilized as an alternative method because of their capacity for fast and reliable species identification (see Humbert, Quiblier & Gugger, 2010). These techniques include conventional PCR (Hirashita et al., 2000), denaturing gradient gel electrophoresis (DGGE) (Riemann, Steward & Azam, 2000; Rooney-Varga et al., 2005), Restriction fragment length polymorphism (RFLP) (González et al., 2000), fluorescence *in situ* hybridization (FISH) (Morris, Longnecker & Giovannoni, 2006; Teeling et al., 2012), and quantitative PCR (Antonella & Luca, 2013). However, these techniques are limited because only a few algal species can be analyzed at a time and the complex interactions within a microbial community cannot be understood by studying a few dominant species.

The next generation sequencing (NGS) technique is now being regarded because of its capacity to analyze entire community structures of the collected samples at a relatively low cost and over a short period of time. In fact, metatranscriptomic analyses (Gong et al., 2017; Rinta-Kanto et al., 2012) and metagenomic studies (Howard et al., 2011; Li et al., 2011; Yang et al., 2015) present the entire microbial community and biological processes of an algal bloom. These results strongly suggest that total microbial community changes should be analyzed to understand the biological processes driving the algal bloom rather than solely focusing on changes in dinoflagellates.

Most genomic analyses are based on the massive sequencing of amplicons generated by the universal primer set. Since its first development (Weisburg et al., 1991), the 16S universal primer set has been the most widely used because of its broad coverage in microbial community studies (Herlemann et al., 2011; Hunt et al., 2013; Sogin et al., 2006) However, it has often been difficult to analyze changes in phytoplankton communities using the 16S universal primer set due to the outnumbered heterotrophic bacterial sequences in water samples. 16S universal primers do not cover all phytoplankton taxa from cyanobacteria to eukaryotic algae in the 16S rDNA region, and most studies analyze specific taxonomic groups, especially for bacterial communities (Cruaud et al., 2014; Logares et al., 2014; Massana et al., 2015; Valenzuela-González et al., 2016). In order to overcome the difficulty, 18S universal primer set was adopted to increase the coverage and sensitivity for eukaryotic phytoplankton (Bradley, Pinto & Guest, 2016; Stoeck et al., 2010; Tragin, Zingone & Vaulot, 2018). However, cyanobacteria cannot be amplified by the primer set. In a case to analyze phytoplankton species, universal primer sets targeting the plastid 23S rDNA region were designed, but they showed cross-reactivity, amplifying considerable amounts of heterotrophic bacterial sequences as well as those of phytoplankton (Sherwood & Presting, 2007; Yoon et al., 2016). Here, we modified the previously designed universal primer set targeting 23S primers to understand changes in phytoplankton communities from the water samples of algal bloom sites. Modified 23S universal primers presented a much higher specificity for phytoplankton sequences as well as a broader phytoplankton taxon coverage than previous universal primer sets. Using two universal primer sets (16S & 23S universal primers), we compared the community structures in water samples from three sample sites (Bloom, Edge, and Control sites) in Geoje, where HAB occurred in 2015. We also analyzed the correlations between heterotrophic bacteria and phytoplankton to determine the interactions between both groups during the bloom.

## Materials & Methods

### Phytoplankton-specific universal primer set optimized for the MiSeq platform

A universal primer set was designed to increase specificity as well as taxon coverage of phytoplankton (Table 1). A total of 1,473 23S rDNA sequences (997 from proteobacteria and 476 from phytoplankton and cyanobacteria) obtained from the public databases including GenBank (https://www.ncbi.nlm.nih.gov/genbank/) & BOLD (http://www.barcodinglife.org) were compared using the Clustal omega program (http://www.ebi.ac.uk/Tools/msa/clustalo/). A new 23S universal forward primer (P23MISQF1) was designed by several modifications of the previously designed ones (Table S1). Briefly, we added the degenerate sequence at the seventh position from the 5′ end of P23MISQF1 to increase the taxon coverage for the Heterokonts (A/T), which was previously adenine (A) in the A23SrVF1 (Yoon et al., 2016; hereafter referred to as Yoon’s 23S universal primer) and p23SrV-f1 primers (Sherwood & Presting, 2007; hereafter referred to as Sherwood’s 23S universal primer). To increase specificity for phytoplankton, guanine (G) was added to the 3′ end of P23MISQF1 (Table S1). We also introduced two changes in the reverse primer, P23MISQR1 (Table 1). First, the nitrogenous base in the fifth nucleotide from its 5′ end was replaced by pyrimidine (Y) bases to increase the coverage of algal sequences (Table S2). The second modification was the addition of two nucleotides at the 3′ end of P23MISQR1 (Table S2), which increased both its melting temperature (T_m_) and the sequence specificity for phytoplankton sequences. The expected sizes of amplified products by the newly modified 23S universal primer set (hereafter referred to as Kang’s 23S universal primer set) ranged from 407 to 414 bps, which is optimized for the MiSeq platform. In order to evaluate the designed plastid 23S universal primer set, *In-silico* PCR was performed on LSU-132 database (RefNR sequence collection) using the SILVA TestPrime tool with zero mismatches (https://www.arb-silva.de/search/testprime/).

**Table 1:** Primers used in this study.

Primer	5′–3′	Target region	Reference
Bakt_341F	CCTACGGGNGGCWGCAG	16S	Herlemann et al. (2011)
Bakt_805R	GACTACHVGGGTATCTAATCC	16S	
p23SrV_f1	GGA CAG AAAGAC CCT ATG AA	23S	Sherwood & Presting (2007)
p23SrV_r1	TCA GCCTGT TAT CCC TAG AG	23S	
A23SrVF1	GGACARAAAGACCCTATG	23S	Yoon et al. (2016)
A23SrVF2	CARAAAGACCCTATGMAGCT	23S	
A23SrVR1	AGATCAGCCTGT TATCC	23S	
A23SrVR2	TCAGCCTGTTATCCCTAG	23S	
P23MISQF1	GGACARWAAGACCCTATGMAG	23S	(present study)
P23MISQR1	AGATYAGCCTGTTATCCCT	23S	
Nex Bakt_341F	TCG TCG GCA GCG TCA GAT GTG TAT AAG AGA CAG CCT ACG GGN GGC WGC AG	16S	
Nex Bakt_805R	GTC TCG TGG GCT CGG AGA TGT GTA TAA GAG ACA GGA CTA CHV GGG TAT CTA ATC C	16S	
Nex P23MISQF1	TCG TCG GCA GCG TCA GAT GTG TAT AAG AGA CAG GGA CAR WAA GAC CCT ATG MAG	23S	
Nex P23MISQR1	GTC TCG TGG GCT CGG AGA TGT GTA TAA GAG ACA GAG ATY AGC CTG TTA TCC CT	23S	

### Sample collection and DNA extraction

**Figure 1:**
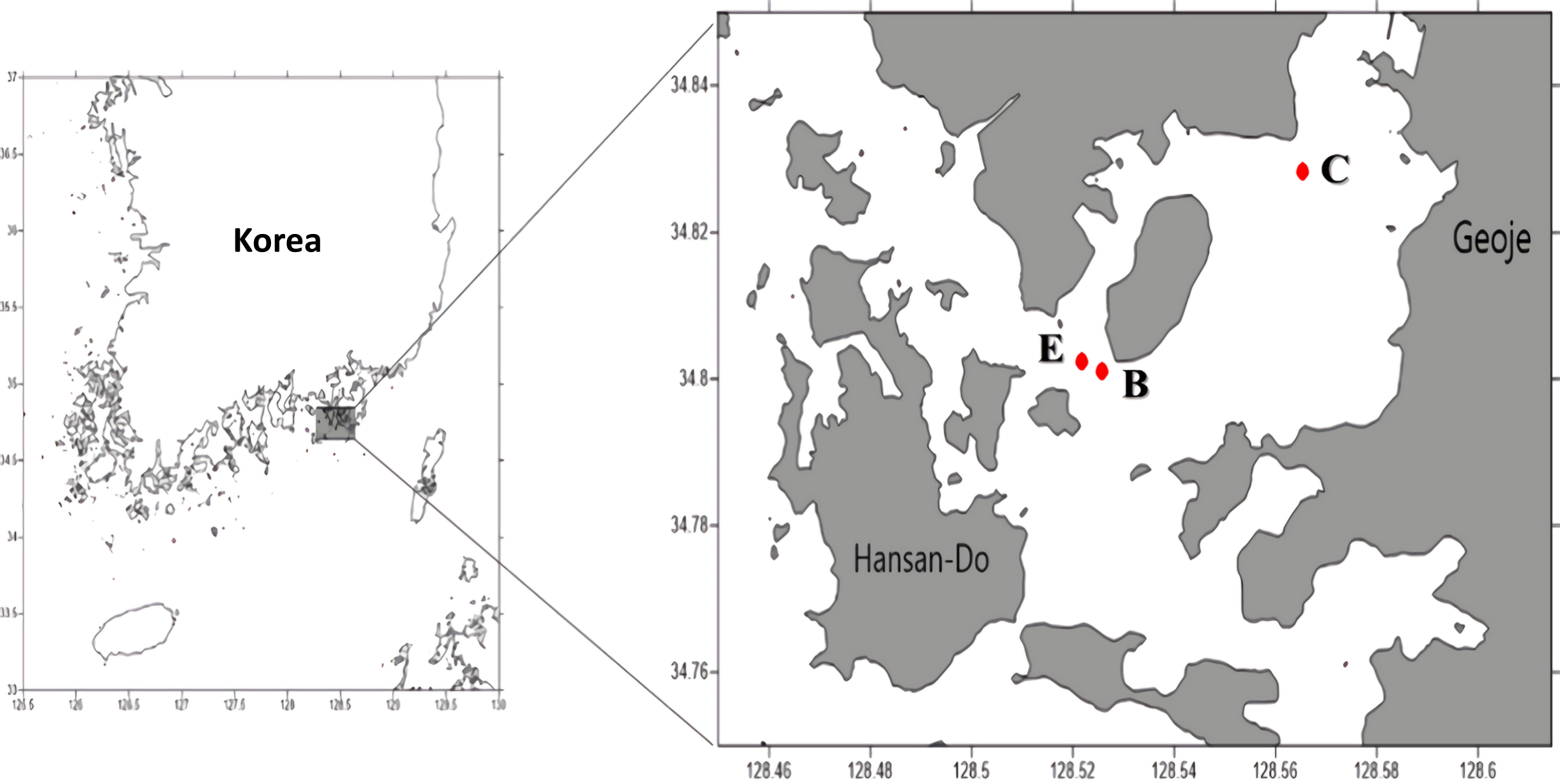
Sampling sites (C: Control site, E: Edge site, B: Bloom site).

We tested the reliability of Kang’s 23S universal primer set using two seawater samples collected from the East/Japan Sea in 2014 as part of the “Long-term change of structure and function in marine ecosystems of Korea” project funded by the Ministry of Oceans and Fisheries, Korea. To analyze the bloom, water samples were collected on Aug 20, 2015 from three sample sites in Geoje, Korea (Fig. 1). A water sample collected from a site distantly located from the bloom (N34°82′825″, E128°56′533″) was used as the control. Two water samples were collected from the center of the bloom (N34°80′110″, E128°52′572″) and at its edge site, which was close to the bloom, but did not exhibit a water-color change (E site; N34°80′245″, E128°52′175″). From each sample site, 1L of surface water was collected and stored in an ice bucket before filtration through a 0.45 µm GH polypro membrane filter (Pall Corporation, New York, NY, USA). The membrane filters were then cut into small pieces using autoclaved dissecting scissors and completely grinded in a mortar and pestle with liquid nitrogen. Genomic DNA was extracted using a DNeasy^®^ plant mini kit (Qiagen, Hilden, Germany), following the manufacturer’s instructions. Isolated genomic DNA was quantified using an ND-1000 nanodrop spectrophotometer (Thermo Scientific, Waltham, MA, USA) and stored at −70 °C until used for library construction.

### Library preparation and sequencing

Isolated genomic DNA was used as a template for the library construction of MiSeq sequencing. Libraries derived from the 16S (Bakt_341F and Bakt_805R) and Kang’s 23S universal primer sets (P23MISQF1 and P23MISQR1) were used for microbial and phytoplankton communities, respectively (Table 1). Additionally, two 23S universal primer sets (Sherwood’s and Yoon’s 23S universal primer sets) were used to test the reliability of Kang’s 23S universal primer set for phytoplankton community analyses (Table 1). The library was constructed using the Nextera XT index kit (Illumina, San Diego, CA, USA) according to the manufacturer’s manual. First, PCR amplification was done using the universal primer sets (NexBakt_341F and NexBakt_805R, NexP23MISQF1 and NexP23MISQR1), which overhang the adapter sequence on forward and reverse primers, respectively (Table 1). The PCR reaction (total volume 20 µL) contained 10 ng of template, 1 µL of each primer (10 pmol), 2 µL of dNTPs (10 mM), 0.2 µL Phusion High Fidelity DNA polymerase (New England Biolabs, Hitchin, UK), and 4 µL 5X buffer. The first PCR condition was an initial denaturation at 94 °C for 3 min, followed by 15 cycles at 94 °C for 30 s, 55 °C for 30 s, and 72 °C for 30 s, with a final extension at 72 °C for 3 min. The PCR products from the first amplification were purified using the AccuPrep^^®^^ PCR purification Kit (Bioneer, Daejeon, Republic of Korea) and eluted with 20 µL of elution buffer. The same conditions including PCR cycles and volume of components were employed for the second PCR amplification, except that 4 µL of purified first PCR product was used as a template and the indexing primers for the MiSeq platform. The second PCR amplicon was separated by 1.5% agarose gel electrophoresis and stained with loading star dye (Dynebio, Seoul, Republic of Korea). PCR products with the expected sizes (approx. 580 bp for analysis of 16s rRNA sequences and approximately 540 bp for analysis of 23s rRNA sequences) were cut from the gel and purified using an AccuPrep^^®^^ gel purification Kit (Bioneer, Daejeon, Republic of Korea). The quality and quantity of the libraries were measured using a 2100 Bioanalyzer (Agilent Technologies, Santa Clara, CA, USA). Finally, constructed libraries were loaded with a MiSeq 600-cycle Reagent Kit v3 (Illumina, San Diego, CA, USA) to perform 300-bp paired-end sequencing on a MiSeq instrument.

### Bioinformatics analysis of NGS data

The raw reads with a low quality (QV < 20) and shorter than 100 nucleotides were eliminated from further analysis using the CLC Genomic Workbench v.8.0 (CLC Bio, Cambridge, MA, USA). The reads were merged with longer than 6 bp overlapping sequences without any mismatches. The merged read with the expected size ranges (400∼500 for 16S and 350∼450 for 23S) were selected and their primer sequences were trimmed using Mothur software v.1.35.0 (Schloss et al., 2009). The obtained merged reads were clustered into operational taxonomic units (OTUs) at 99% similarities and chimeras were removed using UCHIME software v.8.1 (Edgar et al., 2011). Operational taxonomic units (OTUs) with less than 10 merged reads or below 0.1% of the total merged reads were eliminated from further analysis. The species name for each OTU was assigned by the similarity search using a blastn search of BLAST +2.2.30 (Camacho et al., 2009) on the NCBI non-redundant nucleotide database (ftp://ftp.ncbi.nlm.nih.gov/blast/db/; accession date: 04/04/2017). Top-scored species name was assigned for each OTU with higher than 98% sequence identity to the database. The OTUs with 90–98% identities in the database were described as “Genus name with highest score” followed by “sp.” OTUs with less than 90% identity were classified as “Unknown”. A phylogenetic tree was constructed by the Minimum Evolution algorithm using Molecular Evolutionary Genetics Analysis (MEGA ver 6.0) (Tamura et al., 2013).

### Quantitative PCR for microbial communities

To quantify total microorganisms and phytoplankton communities, qPCR with two different universal primer sets (Bakt_341F and Bakt_805R and P23MISQF1 and P23MISQR1, respectively) were employed (Table 1). It was performed using a DNA Engine Chromo 4 Real-Time PCR Detection System (Bio-Rad, Hercules, CA, USA) under the following conditions: initial template denaturation (94 °C for 3 min); 40 amplification cycles (94 °C for 30 s; 55 °C for 30 s; 72 °C for 30 s) and a final extension step 72 °C for 3 min. A 20 µL volume of the qPCR mixture contained 10 µl of 2 X SYBR Green premix Ex Taq II (Takara Bio Inc., Kuratsu, Japan), 4 µl of template, 1 µl of forward and reverse primers (10 pmol), and 4 µl of purified PCR grade water. Standard curves were constructed to confirm the efficiency of each primer set and quantify copy numbers.

## Results

### Comparative analysis of phytoplankton community structures generated by three 23S universal primer sets

As the result of SILVA TestPrime tool, all matched sequences (7,749) were photosynthetic groups without any heterotrophic bacterial sequences indicating high specificity of Kang’s 23S universal primer set to photosynthetic phytoplankton. To determine the reliability of the modified Kang’s 23S universal primer set for the analysis of the phytoplankton community, the NGS results of the same seawater sample with three different 23S universal primer sets (Sherwood’s, Yoon’s, and Kang’s) were compared (Table 2). After trimming and clustering the raw reads, 103,359, 175,854, and 54,129 of the merged reads were obtained by Sherwood’s, Yoon’s, and Kang’s 23S primer sets, respectively (Table 2). The highest OTU numbers were identified in the results of Kang’s primer set (98 OTUs) followed by Yoon’s (81 OTUs) and Sherwood’s primer set (28 OTUs). Only 16 eukaryotic algal OTUs were obtained using Sherwood’s 23S primers, while 60 and 67 phytoplankton OTUs (one cyanobacteria and 59 eukaryotic algae by Yoon’s and one cyanobacteria and 66 eukaryotic algae by Kang’s) were identified, respectively (Table 2). In contrast, the highest heterotrophic bacterial OTUs were identified by Sherwood’s primer set (eight), followed by Yoon’s primer set (six OTUs). Only three heterotrophic bacterial OTUs were identified by Kang’s primer set (Table 2). Proportions of bacterial reads were also highest in Sherwood’s primer set (70.2%) followed by Yoon’s primer (59.34%). Only 0.86% of the bacterial sequences was identified by Kang’s 23S primer set. These results showed that Kang’s 23S universal primer set is a reliable tool for analyzing a phytoplankton community because of its high taxon-specificity, excluding bacterial sequences.

**Table 2:** Comparison of sea water OTUs generated by three 23S universal primer sets (Sherwood’s, Yoon’s, Kang’s).

Phylum	Description	Sherwood’s					Yoon’s					Kang’s (present study)				
		OTU	subtotal	Contigs	Proportion (%) of contigs number	Subtotal (%)	OTU	subtotal	Contigs	Proportion (%) of contigs number	Subtotal (%)	OTU	subtotal	Contigs	Proportion (%) of contigs number	Subtotal (%)
Proteobacteria	Heterotrophic prokaryote	8	8	72,563	70.2	70.2	5	6	104,351	59.34	59.34	3	3	464	0.86	0.86
Verrucomicrobia	Heterotrophic prokaryote						1		8							
Cyanobacteria	Photosynthetic prokaryote						1	1	26	0.01	0.01	1	1	326	0.6	0.6
Bacillariophyta	Photosynthetic eukaryote	7	16	136	0.13	0.23	25	59	9,284	5.28	7.26	27	66	18,366	33.93	87.04
Chlorophyta	Photosynthetic eukaryote	1		17	0.02		7		172	0.1		10		1,952	3.61	
Cryptophyta	Photosynthetic eukaryote						3		21	0.01						
Haptophyta	Photosynthetic eukaryote	4		40	0.04		16		1,364	0.78		20		21,485	39.69	
Miozoa	Photosynthetic eukaryote	3		26	0.03		4		1,543	0.88		4		3,360	6.21	
Ochrophyta	Photosynthetic eukaryote						1		8	0		1		849	1.57	
Rhodophyta	Photosynthetic eukaryote	1		8	0.01		1		327	0.19		1		599	1.11	
Streptophyta	Photosynthetic eukaryote						2		33	0.02		3		503	0.93	
Unknown		4	4	30,569	29.58	29.58	15	15	58,717	33.39		28	28	6,225	11.5	11.5
Total		28	28	103,359	100	100	81	81	175,854	100	67	98	98	54,129	100	100

The taxon coverage of three 23S universal primer sets were also compared (Table 2). Five eukaryotic phytoplankton phyla, Bacillariophyta, Chlorophyta, Haptophyta, Miozoa, and Rhodophyta, were identified by Sherwood’s primer set, while two and three additional eukaryotic phytoplankton phyla were identified by Kang’s (Ochrophyta and Streptophyta) and Yoon’s primer sets (Ochrophyta, Streptophyta, and Cryptophyta), respectively (Table 2). Cyanobacterial sequences were identified only by Yoon’s and Kang’s primer sets. Among the 20 most abundant OTUs, 70.21% (7 OTUs) and 52.36% (3 OTUs) were occupied by the bacterial OTUs of Sherwood’s and Yoon’s primer sets, respectively (Table S3), which was not suitable for phytoplankton community analysis presenting dominant bacterial OTUs. In contrast, only one bacterial OTU ranked at 20th with a negligible proportion (0.76%) by Kang’s 23S universal primer set supporting that Kang’s 23S universal primer set is a reliable tool for analyzing the phytoplankton community structure from the entire microbial community with a high taxon specificity and coverage.

### Changes in total microbial communities during the bloom

**Figure 2:**
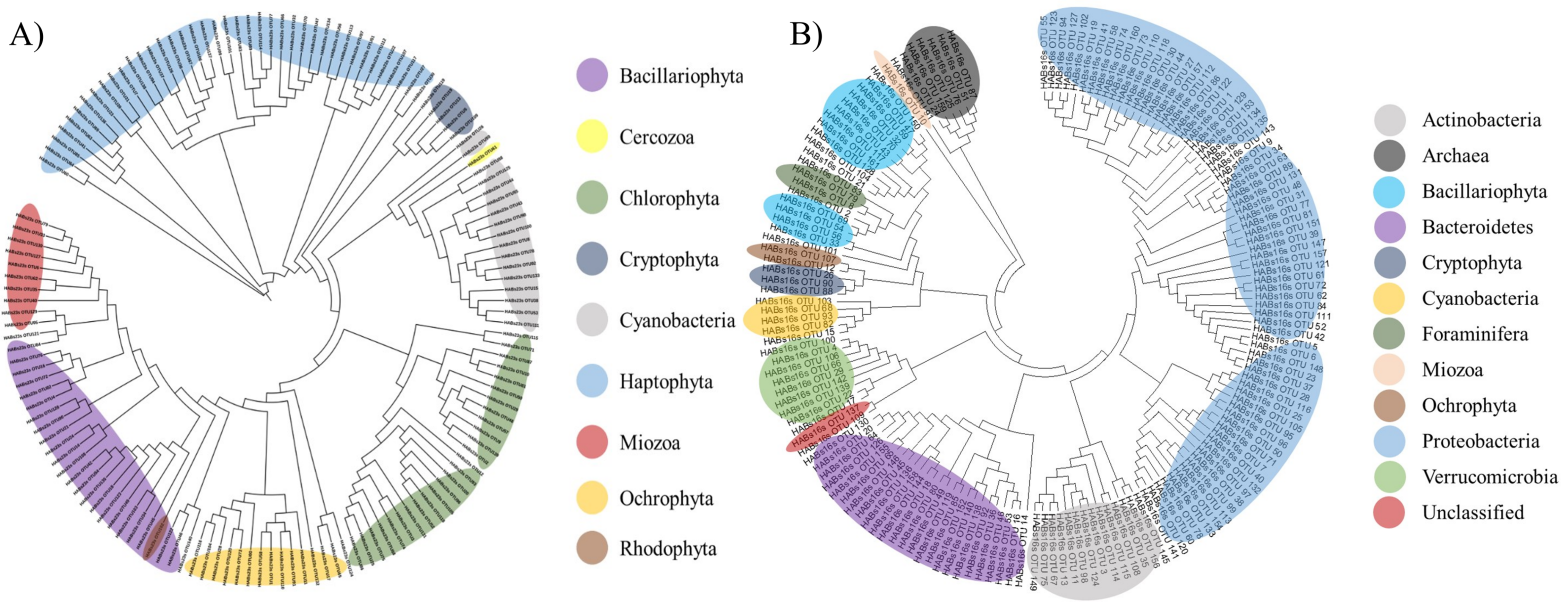
Phylogenetic tree of microbial OTUs generated by 16S universal primer set (A) and by 23S universal primer set (B). Phylogenetic tree was constructed by the Minimum Evolution algorithm using Molecular Evolutionary Genetics Analysis (MEGA ver 6.0).

To determine the microbial community changes caused by algal bloom, an NGS analysis was conducted using the 16S universal primer set (Table 1). After trimming and clustering the raw reads, 6,588 reads from the control station, 21,190 from the edge, and 32,461 from the bloom were generated using the 16S universal primer set (Table 3). Clustered with a 99% sequence identity, 161 microbial OTUs were identified from three water samples in the coastal waters of Geoje in 2015. The highest OTU numbers were identified at the edge (103), followed the bloom (89), and control (61) sites (Table 4). All OTUs showed more than a 98% sequence identity indicating the high quality of the 16S database (Table 3). Of 161 OTUs, 23 OTUs were identified as ‘Uncultured Bacteria’ their phyla were determined by phylogenetic analysis. (Fig. 2A).

**Table 3:** Comparison of taxa levels assigned to OTUs generated by 16S and 23S universal primer set.

	Identity	Control			Edge			Bloom		
		OTUs	Contigs	Proportion (%)	OTUs	Contigs	Proportion (%)	OTUs	Contigs	Proportion (%)
16S	Above 98%	61	6,588	100	103	21,190	100	89	32,461	100
	98% to 90%	–	–	–	–	–	–	–	–	–
	Below 90%	–	–	–	–	–	–	–	–	–
	Total	61	6,588	100	103	21,190	100	89	32,461	100
23S	Above 98%	31	11,294	45.02	26	20,204	64.68	22	1,4907	63.87
	98% to 90%	36	13,791	54.98	54	10,663	34.14	56	8,139	34.87
	Below 90%	0	0	0.00	2	369	1.18	3	295	1.26
	Total	67	25,085	100	82	31,236	100	81	23,341	100

**Table 4:** Summary of OTUs in water samples of red tides produced by 16S universal primer.

	Phylum	Control		Edge		Bloom	
		OTUs	Proportion (%)	OTUs	Proportion (%)	OTUs	Proportion (%)
16S	Archaea	5	2.46	3	0.62	2	0.52
	Actinobacteria	9	16.61	9	8.71	8	7.95
	Bacteroidetes	4	1.20	20	8.82	19	10.73
	Proteobacteria	26	57.39	43	54.96	37	58.23
	Verrucomicrobia	2	2.41	8	14.42	8	13.07
	Foraminifera	7	15.57	1	0.27	1	0.13
	Cyanobacteria	2	0.66	5	3.43	4	3.06
	Bacillariophyta	6	3.70	7	2.42	4	1.01
	Cryptophyta	–	–	2	1.43	2	1.54
	Miozoa	–	–	1	2.38	1	1.90
	Ochrophyta	–	–	2	2.20	2	1.72
	Unclassified	–	–	2	0.34	1	0.14
	Total	61	100	103	100	89	100
23S	Bacillariophyta	11	14.33	13	7.24	13	5.63
	Cercozoa	1	0.32	1	0.28	1	0.30
	Chlorophyta	21	65.87	8	14.74	9	19.34
	Cryptophyta	4	1.72	3	7.71	4	10.78
	Cyanobacteria	3	1.36	11	8.78	13	10.46
	Haptophyta	17	8.44	29	13.96	24	9.70
	Miozoa	9	7.78	6	2.75	4	2.33
	Ochrophyta	1	0.18	8	43.18	9	40.05
	Rhodophyta	–	–	1	0.17	1	0.15
	Unclassified	–	–	2	1.18	3	1.26
	Total	67	100	82	100	81	100

Thirteen ‘uncultured’ OTUs were bacteria, including eight Bacteroidetes, three Proteobacteria, one Actinobacteria, and one Verrucomicrobia, while seven belonged to Archaea and the final one was the eukaryotic algal species, Bacillariophyta. Finally, it was difficult to determine the taxonomic rank of two OTUs (OTU109 and OTU137) by phylogenetic analysis and were therefore named ‘unclassified OTUs’ (Table 4). Besides these two ‘unclassified’ OTUs, the 159 obtained OTUs amplified by the 16S universal primer set were further classified into 11 phyla, including five prokaryotic heterotrophs (Actinobacteria, Bacteroidetes, Proteobacteria, Verrucomicrobia, and Archaea), 1 eukaryotic protist (Foraminifera), one photosynthetic prokaryote (Cyanobacteria), and 4 photosynthetic eukaryote (Mioza, Ochrophyta, Bacillariophyta, and Cryptophyta) (Table 4 and Fig. 2A).

**Figure 3:**
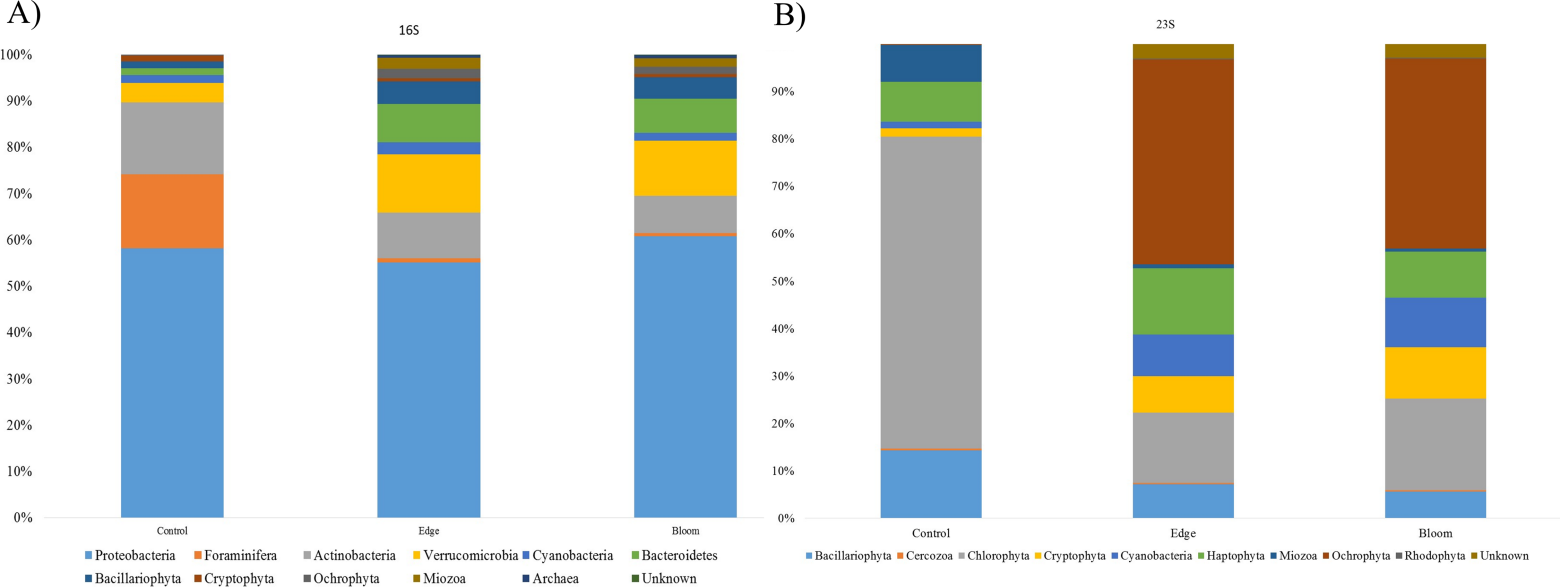
(A) Bacterial community structure at phylum level in red tidal plankton sample; (B) photosynthetic phytoplankton community structure at phylum level in red tidal plankton sample.

Community structures of three sample sites generated by the 16S universal primer set were compared (Table 4). In all three sites, OTUs in Proteobacteria were predominant in all three sites and occupied more than 50% of the total OTU numbers and proportions (Fig. 3A), whereas only 24 photosynthetic phytoplankton OTUs of all the microorganisms (cyanobacteria and eukaryotic algae) were identified and their proportions were very low (4.36% and 11.86% at the control and edge sites, respectively) (Table 4). At the control site, heterotrophic bacterial OTUs occupied 80.07%, followed by the eukaryotic protists, Foraminifera (15.57%), and photosynthetic phytoplankton (4.36%). Unlike the microbial community structure at the control site, those at both the bloom and edge sites were highly similar (Table 4). In both bloom and edge sites, proportions of heterotrophic bacteria were 87.53% and 90.5%, respectively, which was higher than those in the control site (Table 4). In heterotrophic bacteria, proportions of Bacteroidetes and Verrucomicrobia were among the most significantly increased phyla during the bloom occurred; their proportions increased by 7.35 and 5.98 folds at the edge site and 8.94 and 5.42-folds at the bloom site, respectively (Table 4). In contrast, Actinobacteria abundance decreased during the bloom. Among the phytoplankton phyla, Cryptophyta, Miozoa, and Ochrophyta, were only identified at the bloom sites, while the proportions of Bacillariophyta were low at both edge and bloom sites (Table 4). Proportions of phytoplankton at bloom and edge sites were 11.86% and 9.23%, respectively, which was also higher than at the control site (4.36%). Collectively, proportions of both photosynthetic algae and heterotrophic bacteria increased as the bloom occurred. However, proportions of Foraminifera, the amoeboid protist phylum, considerably decreased from 15.57% at the control site to 0.27% and 0.13% at the edge and bloom sites, respectively (Fig. 3 and Table 4).

Operational taxonomic units in Cryptophyta, Ochrophyta and Miozoa, were only identified at the bloom and edge sites (Table 4). Interestingly, all the OTUs exclusively identified at bloom sites were those responsible for algal blooming. One Miozoa (OTU10) at the bloom and edge sites was *Karenia mikimotoi* (GenBank Number: AB027236). Dinoflagellate *K. mikimotoi* is one of the common species responsible for harmful algal bloom (HAB) causing massive fish mortality and human health risks (Anderson et al., 2010; Chen et al., 2011; Gentien et al., 2007). Two Ochrophyta (OTU12, OTU107) were *Heterosigma akashiwo,* which is also a well-known species responsible for HAB as well as *K. mikimotoi* (Nagasaki & Yamaguchi, 1997). Operational taxonomic unit 26 (*Teleaulax amphioxeia*) and OTU88, (Plagioselmis sp.) in Cryptophyta were also known as the Cryptophyta bloom (Seoane et al., 2012; Šupraha et al., 2014).

**Figure 4:**
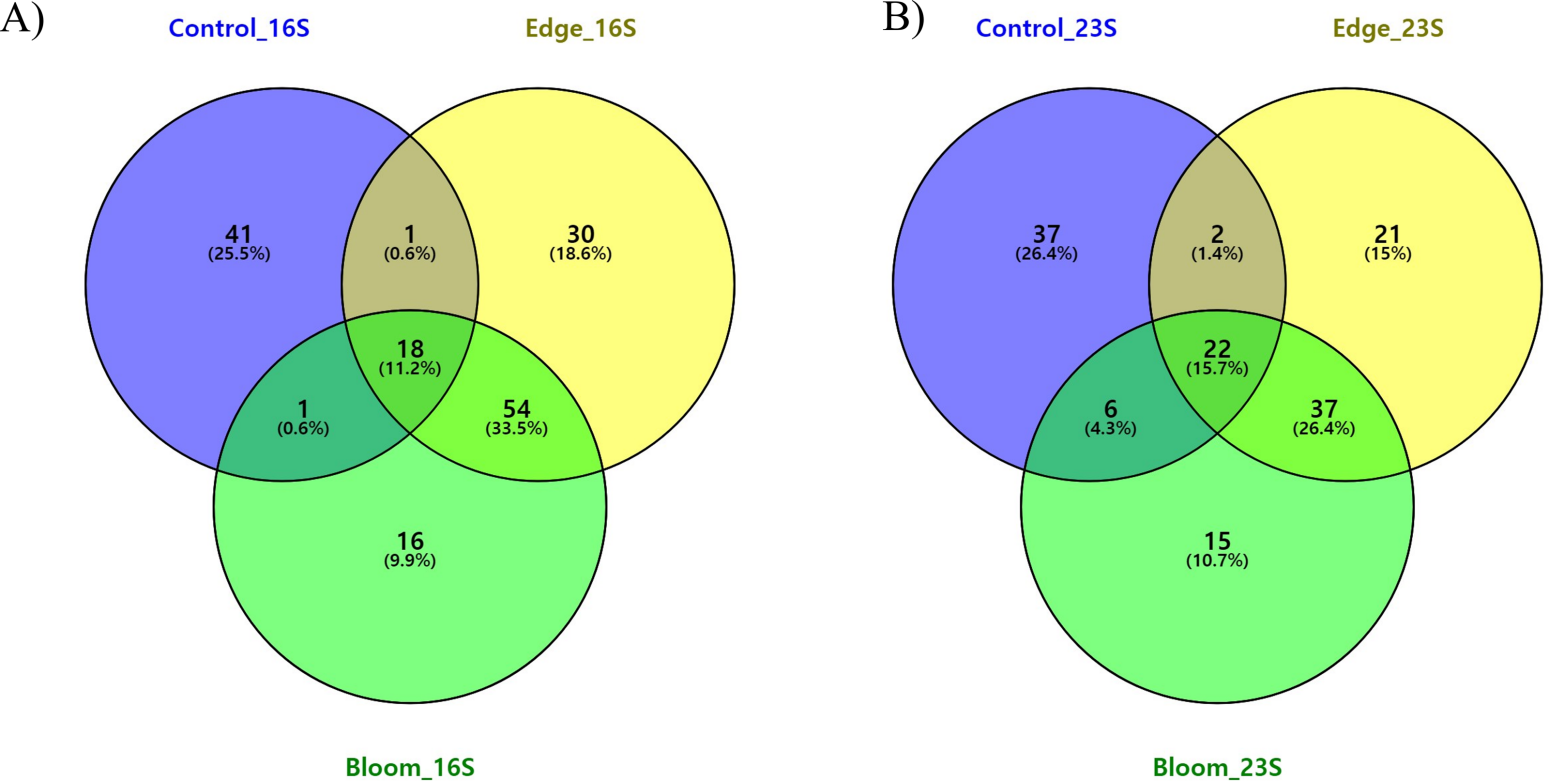
(A) Three-way Venn diagram illustrating the number of shared and unique OTUs obtained by 16S universal primer in red tidal plankton sample; (B) three-way Venn diagram illustrating the number of shared and unique OTUs obtained by 23S universal primer.

To determine the changes in the community structure due to algal bloom, we analyzed the commonly found and bloom-specific OTUs (Table 5, Fig. 4A). Among these 161 OTUs obtained by the 16S universal primer set, 18 OTUs were commonly identified, but their proportions in each site were 64.59%, 64.50%, and 62.93%, respectively (Table 5, Fig. 4A). Except for one cyanobacterial OTU (*Synechococcus* sp., OTU 15), all other commonly identified OTUs were heterotrophic bacterial sequences. *Candidatus pelagibacter* (GenBank: LN850161) was identified as the most abundant OTU in all three sample sites, making up more than 30% of the populations in all three sample sites (Supplementary 5). The control site had the highest site-specific OTU numbers (41), followed by the edge (30), and bloom sites (16). Only one OTU was shared between the control, edge, and bloom sites, whereas there were 54 OTUs supporting community structures of the edge and bloom sites, which were highly similar each other (Table 4 and Supplementary 5). Among the 41 control-specific OTUs, *Virgulinella fragilis* and *Rhodobacteraceae* sp. occupied about 50% of their proportions (Supplementary 4). The other two site-specific OTUs in both bloom and edge sites occupied only small proportions (3.61% and 6.38%, respectively), which suggests that the bloom did not originate from an outbreak of new species, but proportions of preexisting OTUs changed considerably thereby changing the community structure (Table 5).

**Table 5:** Comparison of shared OTUs in three sample sites.

	Site	OTU	Control		Edge		Bloom	
			OTU (%)	Contig (%)	OTU (%)	Contig (%)	OTU (%)	Contig (%)
16S	B-C-E	18	29.51	64.59	17.48	64.50	20.22	62.93
	C-E	1	1.64	0.41	0.97	0.24	–	–
	B-C	1	1.64	0.38	–	–	1.12	0.16
	B-E	54	–	–	52.42	28.88	60.68	33.30
	C	41	67.21	34.62	–	–	–	–
	E	30	–	–	29.13	6.38	–	–
	B	16	–	–	–	–	17.98	3.61
	Total	161	100	100	100	100	100	100
23S	B-C-E	23	34.33	71.19	28.05	72.81	28.40	76.43
	C-E	1	1.49	0.64	1.22	0.14	–	–
	B-C	6	8.96	14.25	–	–	7.4	1.43
	B-E	37	–	–	45.12	22.86	45.68	19.46
	C	37	55.22	13.92	–	–	–	–
	E	21	–	–	25.61	4.19	0.00	–
	B	15	–	–	–	–	18.52	2.68
	Total	140	100	100	100	100	100	100

Operational taxonomic units with more than two-fold changes were analyzed (Table 6). Proportions of 10 OTUs (seven heterotrophic bacteria and three photosynthetic phytoplankton) increased by more than two fold at the edge and bloom sites, respectively, unlike those from the control sites. Moreover, as shown in the community structure, changes in OTUs at the edge and bloom sites were highly similar (Table 6). Increased heterotrophic bacterial OTUs belonged to phyla Bacteroidetes, Verrucomicrobia, and Proteobacteria (Table 6). Although two phytoplankton OTUs, *K. mikimotoi* and *H. akashiwo* were identified in the bloom sites generated by the 16S universal primer set, their proportions were so low due to the outnumbered bacterial sequences (Table 6). Eight OTUs were identified as species that were highly decreased by the bloom (Table 7). The decreased OTUs in both bloom and edge sites were also similar as shown in the increased OTUs. Foraminifera, *V. fragilis* OTUs decreased the most, followed by two bacterial OTUs, Proteobacteria and Actinobacteria (Table 7). Interestingly, one Rhodobacteraceae sp. (GenBank Number: KU382430) increased while the proportions of the other OTU, which showed a 99% identity to KU382430, decreased at bloom sites (Tables 6 and 7).

**Table 6:** OTUs increased more than two folds compared with control site.

	No.	Edge/Control					Bloom/Control				
		OTUs	GenBank No.	Species	Phylum	Fold	OTUs		Species	Phylum	Fold
16S	1	HABs16s_OTU14	KT731620	Uncultured Sphingobacteriales	Bacteroidetes	6.95	HABs16s_OTU16	JF488529	*Bacteroidetes sp.*	Bacteroidetes	7.17
	2	HABs16s_OTU4	HQ675288	*Verrucomicrobia sp.*	Verrucomicrobia	4.93	HABs16s_OTU6^a^	KU382430	*Rhodobacteraceae sp.*	Proteobacteria	5.36
	3	HABs16s_OTU15	KU867931	*Synechococcus sp.*	Cyanobacteria	4.25	HABs16s_OTU14	KT731620	Uncultured Sphingobacteriales	Bacteroidetes	5.04
	4	HABs16s_OTU16	JF488529	*Bacteroidetes sp.*	Bacteroidetes	3.09	HABs16s_OTU15	KU867931	*Synechococcus sp.*	Cyanobacteria	3.86
	5	HABs16s_OTU29	KJ411774	*Verrucomicrobia sp.*	Verrucomicrobia	2.77	HABs16s_OTU4	HQ675288	*Verrucomicrobia sp.*	Verrucomicrobia	3.82
	6	HABs16s_OTU6^a^	KU382430	*Rhodobacteraceae sp.*	Proteobacteria	2.67	HABs16s_OTU29	KJ411774	*Verrucomicrobia sp.*	Verrucomicrobia	3.75
	7	HABs16s_OTU20	JF488593	*Bacteroidetes sp.*	Bacteroidetes	2.51	HABs16s_OTU20	JF488593	*Bacteroidetes sp.*	Bacteroidetes	3.19
	8	HABs16s_OTU31	KU382423	*Pelagibacterales sp.*	Proteobacteria	2.41	HABs16s_OTU31	KU382423	*Pelagibacterales sp.*	Proteobacteria	2.81
	9	HABs16s_OTU10^a^	AB027236	*Karenia mikimotoi*	Miozoa	2.38	HABs16s_OTU10^a^	AB027236	*Karenia mikimotoi*	Miozoa	1.90
	10	HABs16s_OTU12^a^	EU168191	*Heterosigma akashiwo*	Ochrophyta	2.02	HABs16s_OTU17^a^	JF488486	*Verrucomicrobia sp.*	Verrucomicrobia	1.7
23S	1	HABs23s_OTU1	EU168191	*Heterosigma akashiwo*	Ochrophyta	218.44	HABs23s_OTU1	EU168191	*Heterosigma akashiwo*	Ochrophyta	203.37
	2	HABs23s_OTU12	FJ858267	*Micromonas sp.*	Chlorophyta	7.49	HABs23s_OTU13	KP142643	*Teleaulax gracilis*	Cryptophyta	10.16
	3	HABs23s_OTU19	KP142645	*Teleaulax acuta*	Cryptophyta	7.13	HABs23s_OTU19	KP142645	*Teleaulax acuta*	Cryptophyta	7.55
	4	HABs23s_OTU13	KP142643	*Teleaulax gracilis*	Cryptophyta	6.48	HABs23s_OTU12	FJ858267	*Micromonas sp.*	Chlorophyta	6.98
	5	HABs23s_OTU5	KP899713	*Teleaulax amphioxeia*	Cryptophyta	4.16	HABs23s_OTU5	KP899713	*Teleaulax amphioxeia*	Cryptophyta	5.86
	6	HABs23s_OTU41	KJ201907	*Chrysochromulina sp.*	Haptophyta	3.81	HABs23s_OTU8	CP000110	*Synechococcus sp.*	Cyanobacteria	4.27
	7	HABs23s_OTU8	CP000110	*Synechococcus sp.*	Cyanobacteria	3.24	HABs23s_OTU41	KJ201907	*Chrysochromulina sp.*	Haptophyta	3.09
	8	HABs23s_OTU11^a^	EU168191	*Heterosigma sp.*	Ochrophyta	2.84	HABs23s_OTU11^a^	EU168191	*Heterosigma sp.*	Ochrophyta	2.55
	9	HABs23s_OTU14^a^	KJ958482	*Rhizosolenia sp.*	Bacillariophyta	2.21	HABs23s_OTU15^a^	CP006271	*Synechococcus sp.*	Cyanobacteria	2.01

**Notes.**

a OTUs exclusively identified in bloom sites.

**Table 7:** OTUs decreased higher than two folds compared with control site.

	No.	C/E					C/B				
		OTUs		Species	Phylum	Fold	OTUs		Species	Phylum	Fold
16S	1	HABs16s_OTU2^a^	JN207220	*Virgulinella fragilis*	Foraminifera	−11.29	HABs16s_OTU2^a^	JN207220	*Virgulinella fragilis*	Foraminifera	−11.29
	2	HABs16s_OTU5^a^	KU382430	*Rhodobacteraceae sp.*	Proteobacteria	−6.59	HABs16s_OTU5^a^	KU382430	*Rhodobacteraceae sp.*	Proteobacteria	−6.59
	3	HABs16s_OTU24	KT424654	Uncultured marine euryarchaeote	Archaea	−5.15	HABs16s_OTU24	KT424654	Uncultured marine euryarchaeote	Archaea	−4.71
	4	HABs16s_OTU35	LC094544	*Microbacteriaceae sp.*	Actinobacteria	−4.74	HABs16s_OTU11	JF488172	*Actinobacterium sp.*	Actinobacteria	−3.40
	5	HABs16s_OTU11	JF488172	*Actinobacterium sp.*	Actinobacteria	−4.01	HABs16s_OTU35	LC094544	*Microbacteriaceae sp.*	Actinobacteria	−3.19
	6	HABs16s_OTU8^a^	JN207229	*Virgulinella fragilis*	Foraminifera	−2.50	HABs16s_OTU8^a^	JN207229	*Virgulinella fragilis*	Foraminifera	−2.50
	7	HABs16s_OTU3	JF488172	*Actinobacterium sp.*	Actinobacteria	−2.16	HABs16s_OTU61	KX250312	*Erythrobacter sp.*	Proteobacteria	−2.42
	8						HABs16s_OTU3	JF488172	*Actinobacterium sp.*	Actinobacteria	−2.26
23S	1	HABs23s_OTU2	KF285533	*Ostreococcus sp.*	Chlorophyta	−22.41	HABs23s_OTU6	KP826904	*Dinophysis acuta*	Miozoa	−29.85
	2	HABs23s_OTU4^a^	KJ958479	*Chaetoceros sp.*	Bacillariophyta	−7.77	HABs23s_OTU4	KJ958479	*Chaetoceros sp.*	Bacillariophyta	−19.71
	3	HABs23s_OTU9	KF285533	*Ostreococcus tauri*	Chlorophyta	−7.07	HABs23s_OTU2	KF285533	*Ostreococcus sp.*	Chlorophyta	−6.56
	4	HABs23s_OTU10	FO082259	*Bathycoccus prasinos*	Chlorophyta	−6.75	HABs23s_OTU25	FN563097	*Micromonas pusilla*	Chlorophyta	−5.56
	5	HABs23s_OTU6^a^	KP826904	*Dinophysis acuta*	Miozoa	−4.48	HABs23s_OTU9	KF285533	*Ostreococcus tauri*	Chlorophyta	−4.61
	6	HABs23s_OTU34	KJ958485	*Thalassiosira weissflogii*	Bacillariophyta	−4.43	HABs23s_OTU10	FO082259	*Bathycoccus prasinos*	Chlorophyta	−4.56
	7	HABs23s_OTU18	KR709240	*Pseudo-nitzschia multiseries*	Bacillariophyta	−4.02	HABs23s_OTU18	KR709240	*Pseudo-nitzschia multiseries*	Bacillariophyta	−2.57
	8	HABs23s_OTU3	FN563097	*Micromonas pusilla*	Chlorophyta	−2.17	HABs23s_OTU3	FN563097	*Micromonas pusilla*	Chlorophyta	−2.03

**Notes.**

a OTUs exclusively identified in bloom sites.

### Changes in phytoplankton community during the bloom

After trimming and clustering, 25,085, 31,236, and 23,341 reads were finally generated by the Kang’s 23S universal primer set at the control, bloom, and edge sites, respectively (Supplementary 4). At 99% sequence identity, 140 OTUs were obtained and no heterotrophic bacterial OTUs were identified suggesting that Kang’s 23S universal primer set is specific for phytoplankton species (Table 4). The quality of the 23S region database was not as good as that of the 16S region in which species names could not be assigned for about 50% of the OTUs generated by Kang’s 23S primer set at 98% sequence identity (Table 3). Therefore, phylum names were assigned for those exhibiting sequence identities between 90% and 98% as in the previous study (Yoon et al., 2016). Of the total OTUs, 2.15% showed a less than 90% sequence identity to the database and were assigned as “Unknown”. The total 140 OTUs were classified into nine phytoplankton phyla including Haptophyta (27.86%), Chlorophyta (18.57%), Bacillariophyta (14.29%), Cyanobacteria (12.14%), Miozoa (11.43%), Ochrophyta (8.57%), Cryptophyta (3.57%), Cercozoa (0.71%), and Rhodophyta (0.71). Operational taxonomic units in four phyla including Haptophyta, Chlorophyta, Cercozoa, and Rhodophyta were identified exclusively in the results generated by Kang’s 23S universal primer set, which reinforces its importance for microbial community study (Table 4). The most abundant phytoplankton OTU was *Ostreococcus* sp. (32.78%, GenBank number: KF285533), which belongs to Chlorophyta in the control site. *Heterosigma akashiwo* (GenBank number: EU168191) was the most abundant OTU in both the bloom and edge sites and occupied 38.31% and 35.67% of the total reads, respectively (Supplementary 5).

Community structures of the three sample sites generated by Kang’s 23S universal primer set were compared (Table 4, Fig. 3B). In the control site, OTUs in Chlorophyta occupied 65.87%, followed by those in Bacillariophyta (14.33%) and Haptophyta (8.44%). Considering the low proportions of phytoplankton (4.36%), which included Bacillariophyta (3.70%) and Cyanobacteria (0.66%), the 16S universal primer set was not as efficient as the 23S universal primer set in presenting phytoplankton community structures (Table 4). In both the bloom and edge sites, proportions of Ochrophyta were highest (43.18% for edge and 40.05% for bloom) followed by Chlorophyta (14.74% for edge and 19.34% for bloom). The phytoplankton community structures by Kang’s 23S universal primer set were largely similar between the bloom and edge sites as shown in the microbial community structure created by 16S (Table 4 and Fig. 3).

Among phytoplankton OTUs, the highest difference in Chlorophyta between the control and bloom sites was identified. Chlorophyta occupied 65.87% in control site, whereas 14.74% and 19.34% was shown at the bloom and edge sites, respectively. By contrast, OTUs in Ochrophyta occupied only 0.18% at the control site, whereas its proportion was 43.18% at edge site and 40.05% at bloom site, respectively. The proportions of Cryptophyta and cyanobacteria at the control site were also 4.48-folds and 6.46-folds and 6.27-folds and 7.69-folds higher than those at the edge and bloom sites, respectively (Table 4). Although Miozoa decreased at the edge and bloom sites by 2.83 and 3.34 folds, the proportion of *K. mikimotoi* (OTU17) increased. Besides these changes, another algal bloom species, *Alexandrium affine* (OTU65) was identified only in the bloom site. Three ‘unclassified’ OTUs (OTU27, OTU124, OTU132) were also detected at both the bloom and edge sites, but not the control site (Table 4). Collectively, analysis by Kang’s 23S universal primer set was more sensitive to recent changes in phytoplankton communities during the bloom than those by 16S universal primer set (Table 4).

Commonly identified and site-specific OTUs in all three sites were analyzed (Table 5 & Fig. 4B). Among the 140 OTUs obtained by the 23S universal primer set, 23 OTUs were commonly identified in all three sites at 71.19%, 72.81%, and 76.43%, respectively, which was similar to the results obtained by the 16S universal primer set (Table 5). The commonly identified OTUs were eight in Haptophyta, five in Chlorophyta, four in Bacillariophyta, three in Cryptophyta, and one in Cyanobacteria, Cercozoa, and Ochrophyta. Among the 23 commonly identified OTUs, a small chlorophyte, *Ostreococcus* sp. (GenBank numbers: KF285533) was the most abundant species, but was not identified by the 16S universal primer set (Supplementary 6). From the 16S universal primers, the control site exhibited the highest site-specific phytoplankton OTU numbers (37), followed by the edge (21) and bloom sites (15). As shown in the results of the 16S universal primer set, more than 95% of the reads shared between the edge and bloom sites by the 23S universal primer set (Table 5 and Fig. 4B). Control-specific OTUs occupied about 14% of their proportions and the other two site-specific OTUs occupied only marginal proportions (Table 5).

To determine the changes in the phytoplankton community structure caused by the algal bloom, OTUs with more than two-fold changes were analyzed (Table 6). At the edge and bloom sites, nine OTUs were significantly higher than in the control site (Table 6). With the exception of four OTUs, all highly increased phytoplankton OTUs generated by Kang’s 23S primer set were responsible for the algal bloom in the ocean (Table 6). *Heterosigma akashiwo* was identified as the most highly increased phytoplankton species in both the bloom and edge sites, whose proportions were more than 200-fold higher than those in the control site. This result indicated that the major bloom species in the Geoje in 2015 was *H. akashiwo*. Additionally, we identified changes in other species responsible for the bloom including three *Teleaulax* spp. and *K. mikimotoi.* Interestingly, two OTUs in the genus *Micromonas* exhibited a different pattern in which one OTU increased while the other one decreased (Tables 6 and 7). Among the eight OTUs that decreased, chlorophytes including *Ostreococcus* sp. were the most highly decreased OTUs in edge site, followed by *Chaetoceros* sp. (Table 7). Interestingly, *Dinophysis acuta* was the most highly decreased OTU at the bloom site unlike at the control site, and is also known as the species partly responsible for HAB (Table 7).

## Discussion

In this study, we compared the community structures of three sample sites (control, bloom, and edge sites) using MiSeq sequencing platform generated by two different universal primer sets, the 16S (Herlemann et al., 2011) and Kang’s 23S universal primer sets, a newly modified 23S universal primer set was used in this study. Kang’s 23S universal primer set exhibited a specificity for phytoplankton taxa as well as wide coverage from prokaryotic cyanobacteria to eukaryotic algae within phytoplankton taxa (Table 2 and Fig. 2B). Both 16S and Kang’s 23S universal primers successfully presented the community structures of each target taxa during the bloom with little conflicting results. However, there were a few differences between the results of the two universal primer sets. First, we identified that several OTUs in phylum Chlorophyta were not presented by the 16S universal primer set in this study (Table 4 and Fig. 3). In contrast, OTUs in phylum Chlorophyta occupied 65.87% of the total phytoplankton reads in the control site by Kang’s 23S universal primer set (Table 4). As one of the smallest photosynthetic picoprasinophytes, *Ostreococcus sp.* and *Micromonas pusilla* are important components of the microbial community in coastal waters (Chrétiennot-Dinet et al., 1995; Countway & Caron, 2006; Worden, 2006). The proportions of *Ostreococcus* sp. decreased by 22.41 folds as the bloom occurred (Table 7). Decreased proportions of those picoparsinophytes may be one of the potential markers for early detection of the bloom by *H. akasiwo*, but additional studies should be made. Second, Kang’s 23S universal primer set not only presented higher numbers of phytoplankton phyla, but also exhibited clear proportional changes (Table 4 and Fig. 3). Although we were able to detect algal OTUs responsible for the bloom by the 16S universal primer set, their proportions were too low to compare between the control and bloom sites. However, Kang’s 23S universal primer set could detect 200-fold changes during the bloom, which means that this primer set was much more sensitive than the 16S primer set in terms of detecting changes in phytoplankton species during various aquatic events, including the bloom, eutrophication, or other ecological transitions. Alternatively, 18S universal primer set can be used to detect changes in eukaryotic algal species (Bradley, Pinto & Guest, 2016; Pearman et al., 2016; Stoeck et al., 2010; Tragin, Zingone & Vaulot, 2018). Recently 18S universal primer successfully amplified the eukaryotic algae with high specificity without bacterial sequences. However, 18S universal primer cannot amplify cyanobacterial sequences and 23S universal primer appear to be more suitable to analyze phytoplankton community. Although 16S universal primer set failed to present picoparsinophytes, it was also useful in detecting decreases in Foraminifera as the bloom occurred (Fig. 3A). Although species in Foraminifera are amoeboid protists, which are not a target species of Kang’s 23S universal primer set, we were able to determine that these species decreased during the bloom (Table 4). In addition, data generated by the 16S universal primer set were also useful for identifying changes in the bacterial community as the algal bloom proceeded (Table 4 and Fig. 3A). One disadvantage of using Kang’s 23S universal primer set may be the limited plastid 23S sequences in the database. Compared with 5,616,941 small subunit (SSU) data, only 735,238 large subunits (LSU) are currently stored in SILVA (https://www.arb-silva.de/). For this reason, the potential inaccuracy in taxonomic annotation may be possible with the current database. Recent advancements in the NGS platform enabled researchers to supplement the database at a relatively low cost and its quality would be improved in a short time. As the two universal primer sets had both strong and weak points, genomic analyses using both primer sets provided a higher-quality of information than using a single primer set would have. Moreover, we were able to obtain quantitative information from Kang’s 23S universal primer set, which exclusively amplified phytoplankton species. Since higher variety of copy numbers in plastid DNA than those of ribosomal DNA, obtained copy numbers by 23S universal primers may not be far from the real cell numbers (Shi et al., 2011). However, Kang’s 23S universal primer can amplify exclusively photosynthetic phytoplankton sequences, this may at least represent relative quantity of phytoplankton compared with heterotrophic bacterial population in the same sample. In fact, we measured the copy numbers of all the microorganisms (16S universal primer set) and phytoplankton (Kang’s 23S universal primer set) using qPCR. From the data, we estimated the ratio between heterotrophic bacteria (copy numbers by 16S - copy numbers by 23S) and photosynthetic phytoplankton (copy numbers by 23S). This result showed that the ratios of phytoplankton to heterotrophic bacteria in the bloom (0.0410422) and edge sites (0.0483856), which was approximately twofold higher than in the control site (0.0910008). This result supports the previous result HAB redirects carbon and energy flow within the pelagic food-web toward heterotrophic bacteria-dominated processes, primarily through the inhibition of algal growth and enhancement of bacterial proliferation (Šulčius et al., 2017). Since microbial ecosystems in aquatic environments are the result of complicated interactions between photosynthetic phytoplankton species and heterotrophic microorganisms (Carrillo, Medina-Sánchez & Villar-Argaiz, 2002; Cole, 1982; Rooney-Varga et al., 2005), it is often necessary to analyze the phytoplankton community separate from the other bacterial species. Thus, the comparative analysis of the microbial community structures using two universal primer sets (16S and Kang’s 23S) would provide useful information necessary for understanding changes in the microbial community due to various ecological events, including HAB.

As a result of the genomic analysis using two universal primer sets, we were able to make several conclusions about the bloom of *H. akasiwo*. First, the number of *H. akashiwo* during the HAB was high enough to detect even in the control site. Although proportions of *H. akashiwo* increased by over 200-folds as bloom occurred, its numbers were high enough to detect in the control site as well (Table 6). Second, microbial community structures in both the bloom and edge sites were largely similar indicating a strong correlation among different microbial components. In this study, *H. akashiwo* was a strong positive correlation between the red-tide species, such as *K. mikimotoi* and *Teleaulax amphioxeia* (Table 8). *Karenia mikimotoi* showed a strong positive correlation with cyanobacteria, such as *Synechococcus* sp., *H. akashiwo,* and *T. amphioxeia,* which are responsible for HAB, but a strong negative correlation with foraminifera and some Chlorophyta, Miozoa and Bacillariophyta. It is noteworthy that proportions of the other algal bloom species increased during the bloom in addition to a high degree of increase in *H. askashiwo* (Table 6). There have been several reports about blooming with multiple algal species (Tang et al., 2006; Trainer et al., 2010). This may have been due to similar environmental conditions, which were favorable to those responsible for the bloom. However, their proliferation was not as abundant as that of *H. akashiwo* suggesting that the microbial community was not as favorable for these species as they were for *H. akashiwo*. Further studies should be conducted to determine the changes in different HABs, which may be caused by the different algal species. In addition to the increased HAB species, there was a strong correlation between *H. akashiwo* and verrucomicrobia bacteria (Table 8). Although Verrucomicrobia is one of the common bacteria found in soil, fresh, and marine waters, knowledge of biological functions of species in this phyla remains insufficient. The importance of increased Verrucomicrobial OTUs needs to be researched further.

**Table 8:** Species with high degree of correlation with *Heterosigma akashiwo*.

Positively correlated species					
Species	GenBank No.	Correlation	Regression equation	*R*^2^	*P*
*Alpha proteobacterium sp._16S*	HQ675244	0.960254	*y* = 0.1014*x* + 1.1124	0.9221	<0.05
*Bacteroidetes sp._16S*	JF488529	0.909463	*y* = 0.1227*x* + 0.0949	0.8271	<0.05
*Formosa sp._16S*	CP017259	0.980188	*y* = 0.0421*x* + 0.0144	0.9608	<0.01
*Uncultured Sphingobacteriales 16S*	KT731620	0.979752	*y* = 0.047*x* − 0.0162	0.9599	<0.01
*Karenia mikimotoi_16S*	AB027236	0.99239	*y* = 0.0579*x* − 0.0122	0.9848	<0.05
*Roseobacter sp._16S*	KX467571	0.968902	*y* = 0.0323*x* + 0.0139	0.9388	<0.05
*Synechococcus sp._16S*	KU867931	0.991593	*y* = 0.0666*x* − 0.0147	0.9833	<0.01
*Teleaulax amphioxeia_16S*	KP899713	0.990399	*y* = 0.0333*x* + 0.0079	0.9809	<0.01
*Verrucomicrobia sp._16S*	HQ675288	0.999391	*y* = 0.3042*x* + 2.3955	0.9988	<0.01

Species with a high degree of correlation were considered candidate marker species in the bloom caused by *H. akashiwo*. However, different microbial community structures are expected in blooms by either different bloom species or environmental conditions. As more data is accumulated, we will acquire more reliable marker species, which could be used as a warning system against HAB.

## Conclusion

In conclusion, microbial community structures of harmful algal bloom (HAB) caused by *Heterosigma akashiwo* were analyzed by NGS platform. Comparative analysis of data generated by two universal primer sets (16S and 23S) provided useful information about the changes in the community during the *H. akashiwo* bloom, including the ratio between phytoplankton and total microbiome and the correlations between various microbial species. These results suggested that algal blooms occur because of complicated interactions between microbial components, directly or indirectly, and more genomic information produced by the two different universal primer sets is necessary to diagnose or forecast HABs.

##  Supplemental Information

10.7717/peerj.4854/supp-1 Supplemental Information 1 Metagenomic raw data of red-tide water bloomed in Geoje 16S_bloom
16S_control
16S_edge
23S_bloom
23S_control
23S_edge

10.7717/peerj.4854/supp-2 Table S1 Comparison of plastid 23S universal forward primer region

10.7717/peerj.4854/supp-3 Table S2 Comparison of plastid 23S universal reverse primer region

10.7717/peerj.4854/supp-4 Table S3 Top 20 OTUs in East/Japan Sea water sample generated by three 23S universal primer set

10.7717/peerj.4854/supp-5 Table S4 Top 20 OTUs in red-tidal water generated by 16S universal primer set

10.7717/peerj.4854/supp-6 Table S5 Top 20 OTUs in red-tidal water generated by 23S universal primer set

10.7717/peerj.4854/supp-7 Table S6 OTU richness, evenness, Shannon diversity index and Simpson diversity index of sampling sites based on OTU proportion

## Additional Information and Declarations

### Data Availability

The following information was supplied regarding data availability: The raw data are provided as a Supplemental File.
