# Supplementary material for: Genomic analysis of red-tide water bloomed with Heterosigma akashiwo in Geoje
Source: PeerJ. 2018 May 29;6:e4854. doi: 10.7717/peerj.4854 (PMC5983014; doi:10.7717/peerj.4854)
Supplement: Table S1 [file peerj-06-4854-s002.docx]

| Phylum | N | P23SrV_f1  (Sherwood & Presting, 2007) | 5' | G | G | A | C | A | G | A | A | A | G | A | C | C | C | T | A | T | G | A | A | - |  |  | 3’ |
| --- | --- | --- | --- | --- | --- | --- | --- | --- | --- | --- | --- | --- | --- | --- | --- | --- | --- | --- | --- | --- | --- | --- | --- | --- | --- | --- | --- |
|  |  | P23MISQF1  (present study) | 5' | G | G | A | C | A | R | W | A | A | G | A | C | C | C | T | A | T | G | M | A | G |  |  | 3’ |
|  |  | A23SrVF1  (Yoon et al., 2016) | 5’ | G | G | A | C | A | R | A | A | A | G | A | C | C | C | T | A | T | G |  |  |  |  |  | 3’ |
|  |  | A23SrVF2  (Yoon et al., 2016) | 5’ |  |  |  | C | A | R | A | A | A | G | A | C | C | C | T | A | T | G | M | A | G | C | T | 3’ |
| Cercozoa | 3 |  |  | G | G | A | C | A | G | A | A | A | G | A | C | C | C | T | A | T | G | A | A | G | C | T |  |
|  |  |  |  | 3 | 3 | 3 | 3 | 3 | 3 | 3 | 3 | 3 | 3 | 3 | 3 | 3 | 3 | 3 | 3 | 3 | 3 | 3 | 3 | 3 | 3 | 3 |  |
| Chlorophyta | 127 |  |  | G | G | A | C | A | R  A/G | A | A | A | G | A | C | C | C | T | R  A/G | T | G | M  A/C | A | G | Y | T |  |
|  |  |  |  | 127 | 127 | 127 | 127 | 127 | 1/126 | 127 | 127 | 127 | 127 | 127 | 127 | 127 | 127 | 127 | 126/1 | 127 | 127 | 126/1 | 127 | 127 | 125/2 | 127 |  |
| Cryptophyta | 17 |  |  | G | G | A | C | A | G | A | A | A | G | A | C | C | C | T | A | T | G | A | A | G | C | T |  |
|  |  |  |  | 17 | 17 | 17 | 17 | 17 | 17 | 17 | 17 | 17 | 17 | 17 | 17 | 17 | 17 | 17 | 17 | 17 | 17 | 17 | 17 | 17 | 17 | 17 |  |
| Dinophyta | 7 |  |  | G | G | A | C | A | R  A/G | A | A | A | G | A | C | C | C | T | A | T | G | A | A | G | C | T |  |
|  |  |  |  | 7 | 7 | 7 | 7 | 7 | 1/6 | 7 | 7 | 7 | 7 | 7 | 7 | 7 | 7 | 7 | 7 | 7 | 7 | 7 | 7 | 7 | 7 | 7 |  |
| Euglenozoa | 116 |  |  | G | G | A | C | A | G | A | A | A | G | A | C | C | Y  C/T | T | A | Y  C/T | R  A/G | V  A/C/G | A | G | Y  C/T | T |  |
|  |  |  |  | 116 | 116 | 116 | 116 | 116 | 116 | 116 | 116 | 116 | 116 | 116 | 116 | 116 | 115/1 | 116 | 116 | 1/115 | 1/115 | 114/1/1 | 116 | 116 | 115/1 | 116 |  |
| Glaucophyta | 3 |  |  | G | G | A | C | A | G | A | A | A | G | A | C | C | C | T | A | T | G | A | A | G | C | T |  |
|  |  |  |  | 3 | 3 | 3 | 3 | 3 | 3 | 3 | 3 | 3 | 3 | 3 | 3 | 3 | 3 | 3 | 3 | 3 | 3 | 3 | 3 | 3 | 3 | 3 |  |
| Haptophyta | 7 |  |  | G | G | A | C | A | R  A/G | A | A | A | G | A | C | C | C | T | A | T | G | A | A | G | C | T |  |
|  |  |  |  | 7 | 7 | 7 | 7 | 7 | 4/3 | 7 | 7 | 7 | 7 | 7 | 7 | 7 | 7 | 7 | 7 | 7 | 7 | 7 | 7 | 7 | 7 | 7 |  |
| Heterokonta | 97 |  |  | G | G | A | C | A | G | W  A/T | A | A | G | A | C | C | C | T | A | T | G | A | A | G | C | T |  |
|  |  |  |  | 97 | 97 | 97 | 97 | 97 | 97 | 41/56 | 97 | 97 | 97 | 97 | 97 | 97 | 97 | 97 | 97 | 97 | 97 | 97 | 97 | 97 | 97 | 97 |  |
| Rhodophyta | 29 |  |  | G | G | A | C | A | G | A | A | A | G | A | C | C | C | T | A | T | G | A | A | G | C | T |  |
|  |  |  |  | 29 | 29 | 29 | 29 | 29 | 29 | 29 | 29 | 29 | 29 | 29 | 29 | 29 | 29 | 29 | 29 | 29 | 29 | 29 | 29 | 29 | 29 | 29 |  |
| Cyanobacteria | 70 |  |  | G | G | A | C | A | G | A | A | A | G | A | C | C | C | Y  C/T | A | T | G | A | A | G | C | T |  |
|  |  |  |  | 70 | 70 | 70 | 70 | 70 | 70 | 70 | 70 | 70 | 70 | 70 | 70 | 70 | 70 | 1/69 | 70 | 70 | 70 | 70 | 70 | 70 | 70 | 70 |  |
| Alphaproteobacteria | 233 |  |  | A | G | A | C | G | R  A/G | A | V  A/C/G | A | G | A | C | C | C | Y  C/T | R  A/G | T | G | M  A/C | A | C | C | T |  |
|  |  |  |  | 233 | 233 | 233 | 233 | 233 | 2/231 | 233 | 230/1/2 | 233 | 233 | 233 | 233 | 233 | 233 | 196/37 | 55/178 | 233 | 233 | 49/184 | 233 | 233 | 233 | 233 |  |
| Betaproteobacteria | 128 |  |  | A | G | A | C | G | G | A | A | A | G | A | C | C | C | C | R  A/G | T | G | M  A/C | A | C | C | T |  |
|  |  |  |  | 128 | 128 | 128 | 128 | 128 | 128 | 128 | 128 | 128 | 128 | 128 | 128 | 128 | 128 | 128 | 113/15 | 128 | 128 | 127/1 | 128 | 128 | 128 | 128 |  |
| Deltaproteobacteria | 57 |  |  | A | G | A | C | R  A/G | G | A | A | A | G | A | C | C | C | Y  C/T | G | T | G | V  A/C/G | A | C | C | T |  |
|  |  |  |  | 47 | 47 | 47 | 47 | 4/43 | 47 | 47 | 47 | 47 | 47 | 47 | 47 | 47 | 47 | 41/6 | 47 | 47 | 47 | 31/14/2 | 47 | 47 | 47 | 47 |  |
| Epsilonproteobacteria | 74 |  |  | A | G | A | C | G | G | A | R  A/G | A | G | A | C | C | C | C | G | T | G | S | A | C | C | T |  |
|  |  |  |  | 74 | 74 | 74 | 74 | 74 | 74 | 74 | 69/5 | 74 | 74 | 74 | 74 | 74 | 74 | 74 | 74 | 74 | 74 | 14/60 | 74 | 74 | 74 | 74 |  |
| Gammaproteobacteria | 505 |  |  | A | G | A | C | G | G | A | A | A | G | A | C | C | C | C | R  A/G | T | G | M  A/C | A | C | C | T |  |
|  |  |  |  | 504 | 504 | 504 | 504 | 504 | 504 | 504 | 504 | 504 | 504 | 504 | 504 | 504 | 504 | 504 | 1/503 | 504 | 504 | 490/14 | 504 | 504 | 504 | 504 |  |

Supplementary 1. Comparison of plastid 23S universal forward primer region
