# Supplementary material for: Genomic analysis of red-tide water bloomed with Heterosigma akashiwo in Geoje
Source: PeerJ. 2018 May 29;6:e4854. doi: 10.7717/peerj.4854 (PMC5983014; doi:10.7717/peerj.4854)
Supplement: Table S2 [file peerj-06-4854-s003.docx]

Supplementary 2. Comparison of plastid 23S universal reverse primer region

| Phylum | N | P23SrV_r1  (Sherwood & Presting, 2007) | 5' | C | T | | C | T | A | G | G | G | A | T | A | A | C | A | G | G | C | T | G | A | - | - |  | 3’ |
| --- | --- | --- | --- | --- | --- | --- | --- | --- | --- | --- | --- | --- | --- | --- | --- | --- | --- | --- | --- | --- | --- | --- | --- | --- | --- | --- | --- | --- |
|  |  |  | 3’ | G | A | | G | A | T | C | C | C | T | A | T | T | G | T | C | C | G | A | C | T |  |  |  | 5’ |
|  |  | P23MISQR1  (present study) | 5' | - | - | | - | - | A | G | G | G | A | T | A | A | C | A | G | G | C | T | R | A | T | C | T | 3’ |
|  |  |  | 3’ |  |  | |  |  | T | C | C | C | T | A | T | T | G | T | C | C | G | A | Y | T | A | G | A | 5’ |
|  |  | A23SrVR1  (Yoon et al., 2016) | 5’ |  |  | |  |  |  |  | G | G | A | T | A | A | C | A | G | G | C | T | G | A | T | C | T | 3’ |
|  |  |  | 3’ |  |  | |  |  |  |  | C | C | T | A | T | T | G | T | C | C | G | A | C | T | A | G | A | 5’ |
|  |  | A23SrVR2  (Yoon et al., 2016) | 5’ |  |  | | C | T | A | G | G | G | A | T | A | A | C | A | G | G | C | T | G | A |  |  |  | 3’ |
|  |  |  | 3’ |  |  | | G | A | T | C | C | C | T | A | T | T | G | T | C | C | G | A | C | A |  |  |  | 5’ |
| Cercozoa | 3 |  |  | C | | T | C | T | A | G | G | G | A | T | A | A | C | A | G | G | C | T | G | A | T | C | T |  |
|  |  |  |  | 3 | | 3 | 3 | 3 | 3 | 3 | 3 | 3 | 3 | 3 | 3 | 3 | 3 | 3 | 3 | 3 | 3 | 3 | 3 | 3 | 3 | 3 | 3 |  |
| Chlorophyta | 127 |  |  | C | | K  G/T | C | Y  C/T | A | G | G | G | A | T | A | A | C | A | G | G | C | T | D  A/G/T | A | T | Y  C/T | W  A/T |  |
|  |  |  |  | 127 | | 1/126 | 127 | 1/126 | 127 | 127 | 127 | 127 | 127 | 127 | 127 | 127 | 127 | 127 | 127 | 127 | 127 | 127 | 14/110/3 | 127 | 127 | 124/3 | 1/126 |  |
| Cryptophyta | 17 |  |  | C | | T | C | T | A | G | G | G | A | T | A | A | C | A | G | G | C | T | G | A | T | C | T |  |
|  |  |  |  | 17 | | 17 | 17 | 17 | 17 | 17 | 17 | 17 | 17 | 17 | 17 | 17 | 17 | 17 | 17 | 17 | 17 | 17 | 17 | 17 | 17 | 17 | 17 |  |
| Dinophyta | 7 |  |  | C | | T | C | T | A | G | G | G | A | T | A | A | C | A | G | G | C | T | R  A/G | A | T | C | T |  |
|  |  |  |  | 7 | | 7 | 7 | 7 | 7 | 7 | 7 | 7 | 7 | 7 | 7 | 7 | 7 | 7 | 7 | 7 | 7 | 7 | 1/6 | 7 | 7 | 7 | 7 |  |
| Euglenozoa | 116 |  |  | C | | B  C/G/T | Y  C/T | T | A | G | G | G | A | T | A | A | C | A | G | G | C | T | G | A | T | C | T |  |
|  |  |  |  | 116 | | 1/1/114 | 115/1 | 116 | 116 | 116 | 116 | 116 | 116 | 116 | 116 | 116 | 116 | 116 | 116 | 116 | 116 | 116 | 116 | 116 | 116 | 116 | 116 |  |
| Glaucophyta | 3 |  |  | C | | T | C | T | A | G | G | G | A | T | A | A | C | A | G | G | C | T | G | A | T | C | T |  |
|  |  |  |  | 3 | | 3 | 3 | 3 | 3 | 3 | 3 | 3 | 3 | 3 | 3 | 3 | 3 | 3 | 3 | 3 | 3 | 3 | 3 | 3 | 3 | 3 | 3 |  |
| Haptophyta | 7 |  |  | C | | T | C | T | A | G | G | G | A | T | A | A | C | A | G | G | C | T | G | A | T | C | T |  |
|  |  |  |  | 7 | | 7 | 7 | 7 | 7 | 7 | 7 | 7 | 7 | 7 | 7 | 7 | 7 | 7 | 7 | 7 | 7 | 7 | 7 | 7 | 7 | 7 | 7 |  |
| Heterokonta | 97 |  |  | C | | T | C | T | A | G | G | G | A | T | A | A | C | A | G | G | C | T | R  A/G | A | T | C | T |  |
|  |  |  |  | 97 | | 97 | 97 | 97 | 97 | 97 | 97 | 97 | 97 | 97 | 97 | 97 | 97 | 97 | 97 | 97 | 97 | 97 | 1/96 | 97 | 97 | 97 | 97 |  |
| Rhodophyta | 29 |  |  | C | | T | C | T | A | G | G | G | A | T | A | A | C | A | G | G | C | T | R  A/G | A | T | C | T |  |
|  |  |  |  | 29 | | 29 | 29 | 29 | 29 | 29 | 29 | 29 | 29 | 29 | 29 | 29 | 29 | 29 | 29 | 29 | 29 | 29 | 1/28 | 29 | 29 | 29 | 29 |  |
| Cyanobacteria | 70 |  |  | C | | T | C | T | R  A/G | G | G | G | A | T | A | A | C | A | G | G | C | T | G | A | T | C | T |  |
|  |  |  |  | 70 | | 70 | 70 | 70 | 69/1 | 70 | 70 | 70 | 70 | 70 | 70 | 70 | 70 | 70 | 70 | 70 | 70 | 70 | 70 | 70 | 70 | 70 | 70 |  |
| Alphaproteobacteria | 233 |  |  | C | | K  G/T | C | Y  C/T | R  A/G | G | G | G | A | T | R  A/G | A | C | A | G | G | C | T | K  G/T | A | T | N  A/C/G/T | N  A/C/G/T |  |
|  |  |  |  | 233 | | 184/49 | 233 | 178/55 | 37/196 | 233 | 233 | 233 | 233 | 233 | 231/2 | 233 | 233 | 233 | 233 | 233 | 233 | 233 | 230/3 | 233 | 233 | 36/36/149/12 | 152/26/7/48 |  |
| Betaproteobacteria | 128 |  |  | C | | K  G/T | C | Y  C/T | G | G | G | G | A | T | A | A | C | A | G | G | C | G | V  A/C/G | A | T | W  A/T | C |  |
|  |  |  |  | 128 | | 1/127 | 128 | 15/113 | 128 | 128 | 128 | 128 | 128 | 128 | 128 | 128 | 128 | 128 | 128 | 128 | 128 | 128 | 4/1/123 | 128 | 128 | 107/21 | 128 |  |
| Deltaproteobacteria | 57 |  |  | C | | B  C/G/T | C | C | G | G | G | G | A | T | A | A | C | A | G | G | C | G | K  G/T | A | T | C | N  A/C/G/T |  |
|  |  |  |  | 45 | | 2/12/31 | 45 | 45 | 45 | 45 | 45 | 45 | 45 | 45 | 45 | 45 | 45 | 45 | 45 | 45 | 45 | 45 | 17/28 | 45 | 45 | 45 | 2/1/4/38 |  |
| Epsilonproteobacteria | 74 |  |  | C | | S  C/G | C | C | G | G | G | G | A | T | A | A | C | A | G | G | C | T | G | A | T | C | T |  |
|  |  |  |  | 74 | | 60/14 | 74 | 74 | 74 | 74 | 74 | 74 | 74 | 74 | 74 | 74 | 74 | 74 | 74 | 74 | 74 | 74 | 74 | 74 | 74 | 74 | 74 |  |
| Gammaproteobacteria | 505 |  |  | C | | K  G/T | C | Y  C/T | G | G | G | G | A | A | A | A | C | A | G | G | C | T | D  A/G/T | A | T | W  A/T | Y  C/T |  |
|  |  |  |  | 504 | | 14/490 | 504 | 484/20 | 504 | 504 | 504 | 504 | 504 | 504 | 504 | 504 | 504 | 504 | 504 | 504 | 504 | 504 | 18/485/1 | 504 | 504 | 478/26 | 502/2 |  |
