# Supplementary material for: Genomic analysis of red-tide water bloomed with Heterosigma akashiwo in Geoje
Source: PeerJ. 2018 May 29;6:e4854. doi: 10.7717/peerj.4854 (PMC5983014; doi:10.7717/peerj.4854)
Supplement: Table S3 [file peerj-06-4854-s004.docx]

Supplementary 3. Top 20 OTUs in East/Japan Sea water sample generated by three 23S universal primer set

| OTUs | Sherwood’s 23S primer | | | Yoon’s 23S primer | | | | | Kang’s 23S primer | | | | | |
| --- | --- | --- | --- | --- | --- | --- | --- | --- | --- | --- | --- | --- | --- | --- |
|  | Description (GenBank number) | Phylum | (%) | Description (GenBank number) | Phylum | (%) | | Description (GenBank number) | | Phylum | | (%) | |  |
| 1 | Phenylobacterium lituiforme-DQ306696 | Proteobacteria | 62.43 | Phenylobacterium lituiforme - DQ306696.1 | Proteobacteria | | 52.36 | | Leptocylindrus danicus -KC509524 | | Bacillariophyta | | 16.29 | |
| 2 | Unknown | Unknown | 25.6 | Unknown | Unknown | | 31.24 | | Phaeocystis globosa - KC900889 | | Haptophyta | | 15.44 | |
| 3 | Caulobacter segnis -NR076742 | Proteobacteria | 6.14 | Caulobacter segnis - NR076742 | Proteobacteria | | 5.91 | | Chrysochromulina sp. - KJ201907 | | Haptophyta | | 9.45 | |
| 4 | Unknown | Unknown | 3.95 | Leptocylindrus danicus - KC509524.1 | Bacillariophyta | | 2.04 | | Thalassiosira oceanica -GU323224 | | Bacillariophyta | | 5.97 | |
| 5 | Caulobacter sp. - NR076557 | Proteobacteria | 1.41 | Desulfuromonas sp. - CP010802 | Unknown | | 1.97 | | Unknown | | Unknown | | 4.13 | |
| 6 | Rhodoferax saidenbachensis -CP019239 | Proteobacteria | 0.13 | Caulobacter sp. - NR076557 | Proteobacteria | | 1.02 | | Unknown | | Unknown | | 3.29 | |
| 7 | Leptocylindrus danicus - KC509524 | Bacillariophyta | 0.07 | Thalassiosira oceanica - GU323224 | Bacillariophyta | | 0.84 | | Kryptoperidinium foliaceum - GU591328 | | Miozoa | | 3.01 | |
| 8 | Ralstonia mannitolilytica -CP011257 | Proteobacteria | 0.05 | Cerataulina daemon -KJ958484 | Bacillariophyta | | 0.44 | | Isochrysis galbana - KC788222 | | Haptophyta | | 2.4 | |
| 9 | Phaeocystis globosa - KC900889 | Haptophyta | 0.02 | Thalassiosira pseudonana - EF067921 | Bacillariophyta | | 0.41 | | Cerataulina daemon - KJ958484 | | Bacillariophyta | | 2.33 | |
| 10 | Cerataulina daemon -KJ958484 | Bacillariophyta | 0.02 | Kkryptoperidinium foliaceum - GU591328.1 | Miozoa | | 0.41 | | Dinophysis acuta -KP826904 | | Miozoa | | 1.83 | |
| 11 | Micromonas sp. -FJ858267 | Chlorophyta | 0.02 | Rhizosolenia imbricata -KJ958482.1 | Bacillariophyta | | 0.31 | | Emiliania huxleyi - JN022705 | | Haptophyta | | 1.64 | |
| 12 | Thalassiosira pseudonana - EF067921 | Bacillariophyta | 0.02 | Rhizosolenia imbricata -KJ958482 | Bacillariophyta | | 0.3 | | Heterosigma akashiwo -EU168191 | | Ochrophyta | | 1.57 | |
| 13 | Brevundimonas albl -KT865840.1 | Proteobacteria | 0.02 | Phaeocystis globosa -KC900889 | Haptophyta | | 0.3 | | Rhizosolenia imbricate - KJ958482 | | Bacillariophyta | | 1.46 | |
| 14 | Peridiniopsis niei -JQ639742 | Miozoa | 0.01 | Dinophysis acuta - KP826904 | Miozoa | | 0.26 | | Ostreococcus tauri -KF285533 | | Chlorophyta | | 1.34 | |
| 15 | Rhizosolenia imbricata -KJ958482 | Bacillariophyta | 0.01 | Peridiniopsis niei -JQ639742.1 | Miozoa | | 0.2 | | Rhizosolenia imbricata -KJ958482 | | Bacillariophyta | | 1.34 | |
| 16 | Unknown | Unknown | 0.01 | Neosiphonia japonica - KC782888 | Rhodophyta | | 0.19 | | Thalassiosira pseudonana - EF067921 | | Bacillariophyta | | 1.23 | |
| 17 | Chrysochromulina sp. - KJ201907.2 | Haptophyta | 0.01 | Chaetoceros simplex - KJ958479 | Bacillariophyta | | 0.15 | | Neosiphonia japonica - KC782888 | | Rhodophyta | | 1.11 | |
| 18 | Neosiphonia japonica -KC782888.1 | Rhodophyta | 0.01 | Rhizosolenia imbricate - KJ958482 | Bacillariophyta | | 0.15 | | Codium edule -EF426671 | | Chlorophyta | | 0.98 | |
| 19 | Brevundimonas diminuta KX349741.1 | Proteobacteria | 0.01 | Lithodesmium undulatum - KC509525.1 | Bacillariophyta | | 0.11 | | Karlodinium veneficum - JN039300 | | Miozoa | | 0.96 | |
| 20 | Rhizosolenia imbricata KJ958482.1 | Bacillariophyta | 0.01 | Unknown | Unknown | | 0.07 | | Phenylobacterium lituiforme - DQ306696 | | Proteobacteria | | 0.76 | |
