# Supplementary material for: Genomic analysis of red-tide water bloomed with Heterosigma akashiwo in Geoje
Source: PeerJ. 2018 May 29;6:e4854. doi: 10.7717/peerj.4854 (PMC5983014; doi:10.7717/peerj.4854)
Supplement: Table S4 [file peerj-06-4854-s005.docx]

Supplementary 4. Top 20 OTUs in red-tidal water generated by 16S universal primer set

| NO. | Control | | | | | Edge | | | | | Bloom | | | | |
| --- | --- | --- | --- | --- | --- | --- | --- | --- | --- | --- | --- | --- | --- | --- | --- |
|  | Specices | GenBaK  NO. | Proportion  (%) | Phylum |  | Specices | GenBaK  NO. | Proportion  (%) |  |  | Specices | GenBaK  NO. | Proportion  (%) | Phylum |  |
| 1 | Candidatus_pelagibacter sp. | LN850161 | 38.75 | Proteobacteria | PH | Candidatus_pelagibacter sp. | LN850161 | 32.09 | Proteobacteria | PH | Candidatus_pelagibacter sp. | LN850161 | 32.82 | Proteobacteria | PH |
| 2 | Virgulinella fragilis | JN207220 | 11.29 | Foraminifera | PH | Verrucomicrobia sp. | HQ675288 | 10.25 | Verrucomicrobia | PH | Verrucomicrobia sp. | HQ675288 | 7.94 | Verrucomicrobia | PH |
| 3 | Actinobacterium sp. | JF488172 | 10.87 | Actinobacteria | EA | Actinobacterium sp. | JF488172. | 5.04 | Actinobacteria | PH | Rhodobacteraceae sp. | KU382430 | 5.36 | Proteobacteria | PH |
| 4 | Rhodobacteraceae sp. | KU382430 | 6.59 | Proteobacteria | EA | Gammaproteobacterium sp. | JF488603 | 2.70 | Proteobacteria | PH | Actinobacterium sp. | JF488172 | 4.81 | actinobacteria | PH |
| 5 | Virgulinella fragilis | JN207229 | 2.50 | Foraminifera | EA | Rhodobacteraceae sp. | KU382430 | 2.67 | Proteobacteria | PH | Gammaproteobacterium sp. | JF488603 | 2.66 | Proteobacteria | PH |
| 6 | Actinobacterium sp. | JF488172 | 2.16 | Actinobacteria | PH | Candidatus_pelagibacter sp. | LN850157 | 2.44 | Proteobacteria | PH | Candidatus_pelagibacter sp. | LN850157 | 1.96 | Proteobacteria | PH |
| 7 | Verrucomicrobia sp. | HQ675288 | 2.08 | Verrucomicrobia | PH | Karenia mikimotoi | AB027236 | 2.38 | Miozoa | EA | Karenia mikimotoi | AB027236 | 1.90 | Miozoa | EA |
| 8 | Gammaproteobacterium sp. | JF488603 | 1.90 | Proteobacteria | PH | Heterosigma akashiwo | EU168191 | 2.02 | Ochrophyta | EA | Bacteroidetes sp. | JF488529 | 1.85 | Bacteroidetes | PH |
| 9 | Candidatus_pelagibacter sp. | LN850157 | 1.72 | Proteobacteria | PH | Actinobacterium sp. | HQ675191 | 2.02 | Actinobacteria | PH | Verrucomicrobia sp. | JF488486 | 1.77 | Verrucomicrobia | PH |
| 10 | Actinobacterium sp. | HQ675191 | 1.59 | Actinobacteria | PH | Uncultured marine euryarchaeote | KT424654 | 2.01 | Archaea | PH | Synechococcus sp. | KU867931 | 1.76 | Cyanobacteria | PA |
| 11 | Pseudo-nitzschia multiseries | KR709240 | 1.46 | Bacillariophyta | EA | Synechococcus sp. | KU867931 | 1.93 | Cyanobacteria | PA | Formosa sp. | CP017259 | 1.72 | Bacteroidetes | PH |
| 12 | Uncultured bacterium | LT720467 | 1.34 | Bacteroidetes | PH | Formosa sp. | CP017259 | 1.47 | Bacteroidetes | PH | Alphaproteobacterium sp. | HQ675244 | 1.65 | Proteobacteria | PH |
| 13 | Candidatus_pelagibacter sp. | LN850151 | 1.31 | Proteobacteria | PH | Alphaproteobacterium sp. | HQ675244 | 1.43 | Proteobacteria | PH | Bacteroidetes sp. | JF488593 | 1.60 | Bacteroidetes | PH |
| 14 | Cerataulina daemon | KJ958484 | 0.97 | Bacillariophyta |  | Verrucomicrobia sp. | JF488486 | 1.38 | Verrucomicrobia | PH | Heterosigma akashiwo | EU168191 | 1.52 | Ochrophyta | EA |
| 15 | Microbacteriaceae sp. | LC094544 | 0.90 | Actinobacteria | PH | Bacteroidetes sp. | JF488593 | 1.26 | Bacteroidetes | PH | Uncultured marine euryarchaeote | KT424654 | 1.45 | Archaea | PH |
| 16 | Roseobacter sp. | KX467571 | 0.83 | Proteobacteria | PH | Teleaulax amphioxeia | KP899713 | 1.20 | Cryptophyta | EA | Actinobacterium sp. | HQ675191 | 1.45 | Actinobacteria | PH |
| 17 | Gammaproteobacterium sp. | HQ675210 | 0.67 | Proteobacteria | PH | Candidatus_pelagibacter sp. | LN850158 | 1.12 | Proteobacteria | PH | Verrucomicrobia sp. | JF488620 | 1.39 | Verrucomicrobia | PH |
| 18 | Candidatus_pelagibacter sp. | LN850156 | 0.67 | Proteobacteria | PH | Roseobacter sp. | KX467571 | 1.09 | Proteobacteria | PH | Roseobacter sp. | KX467571 | 1.36 | Proteobacteria | PH |
| 19 | Virgulinella fragilis | JN207228 | 0.62 | Foraminifera | PH | Verrucomicrobia sp. | JF488620 | 1.02 | Verrucomicrobia | PH | Candidatus_thioglobus singularis | CP006911 | 1.32 | Proteobacteria | PH |
| 20 | Alphaproteobacterium sp. | HQ675244 | 0.61 | Proteobacteria | PH | Rhizosolenia setigera | FJ002228 | 1.00 | Bacillariophyta | EA | Teleaulax amphioxeia | KP899713 | 1.31 | Cryptophyta | EA |

PH : Prokaryotic Heterotroph, PA: Prokaryotic Autotroph, EA: Eukaryotic Autotroph
