# Supplementary material for: Genomic analysis of red-tide water bloomed with Heterosigma akashiwo in Geoje
Source: PeerJ. 2018 May 29;6:e4854. doi: 10.7717/peerj.4854 (PMC5983014; doi:10.7717/peerj.4854)
Supplement: Table S5 [file peerj-06-4854-s006.docx]

Supplementary 5. Top 20 OTUs in red-tidal water generated by 23S universal primer set

| NO. | Control | | | | Edge | | | | Bloom | | | |
| --- | --- | --- | --- | --- | --- | --- | --- | --- | --- | --- | --- | --- |
|  | Specices | GenBaK  NO. | Proportion  (%) | Phylum | Specices | GenBaK  NO. | Proportion  (%) | Phylum | Specices | GenBaK  NO. | Proportion  (%) | Phylum |
| 1 | *Ostreococcus sp.* | KF285533 | 32.78 | Chlorophyta | *Heterosigma akashiwo* | EU168191 | 38.31 | Ochrophyta | *Heterosigma akashiwo* | EU168191 | 35.67 | Ochrophyta |
| 2 | *Micromonas pusilla* | FN563097 | 19.17 | Chlorophyta | *Micromonas pusilla* | FN563097 | 8.82 | Chlorophyta | *Micromonas pusilla* | FN563097 | 9.47 | Chlorophyta |
| 3 | *Chaetoceros sp.* | KJ958479 | 7.77 | Bacillariophyta | *Teleaulax amphioxeia* | KP899713 | 4.79 | Cryptophyta | *Teleaulax amphioxeia* | KP899713 | 6.75 | Cryptophyta |
| 4 | *Dinophysis acuta* | KP826904 | 4.48 | Miozoa | *Chrysochromulina sp.* | KJ201907 | 4.37 | Haptophyta | *Ostreococcus sp.* | KF285533 | 5.00 | Chlorophyta |
| 5 | *Ostreococcus tauri* | KF285533 | 3.58 | Chlorophyta | *Synechococcus complete* | CP000110 | 3.31 | Cyanobacteria | *Synechococcus sp.* | CP000110 | 4.36 | Cyanobacteria |
| 6 | *Chrysochromulina sp.* | KJ201907 | 3.44 | Haptophyta | *Heterosigma sp.* | EU168191 | 2.84 | Ochrophyta | *Chrysochromulina sp.* | KJ201907 | 3.26 | Haptophyta |
| 7 | *Bathycoccus prasinos* | FO082259 | 3.44 | Chlorophyta | *Micromonas sp.* | FJ858267 | 2.78 | Chlorophyta | *Micromonas sp.* | FJ858267 | 2.59 | Chlorophyta |
| 8 | *Chaetoceros didymus* | KJ671835 | 1.91 | Bacillariophyta | *Rhizosolenia sp.* | KJ958482 | 2.21 | Bacillariophyta | *Heterosigma sp.* | EU168191 | 2.55 | Ochrophyta |
| 9 | *Pseudo-nitzschia multiseries* | KR709240 | 1.54 | Bacillariophyta | *Karenia mikimotoi* |  | 1.88 | Miozoa | *Teleaulax gracilis* | KP142643 | 2.43 | Cryptophyta |
| 10 | *Micromonas pusilla* | FN563097 | 1.40 | Chlorophyta | *Synechococcus sp.* | CP006271 | 1.84 | Cyanobacteria | *Synechococcus sp.* | CP006271 | 2.01 | Cyanobacteria |
| 11 | *Chaetoceros simplex* | KJ958479 | 1.38 | Bacillariophyta | *Teleaulax gracilis* | KP142643 | 1.55 | Cryptophyta | *Karenia mikimotoi* |  | 1.63 | Miozoa |
| 12 | *Chrysochromulina sp.* | KJ201907 | 1.34 | Haptophyta | *Ostreococcus sp.* | KF285533 | 1.46 | Chlorophyta | *Rhizosolenia sp.* | KJ958482 | 1.59 | Bacillariophyta |
| 13 | *Teleaulax amphioxeia* | KP899713 | 1.15 | Cryptophyta | *Phaeocystis globosa* | KC900889 | 1.37 | Haptophyta | *Teleaulax acuta* | KP142645 | 1.44 | Cryptophyta |
| 14 | *Micromonas pusilla* | FN563097 | 1.14 | Chlorophyta | *Teleaulax acuta* | KP142645 | 1.36 | Cryptophyta | *Synechococcus sp.* | CT978603 | 1.11 | Cyanobacteria |
| 15 | *Synechococcus complete* | CP000110 | 1.02 | Cyanobacteria | *Rhizosolenia imbricata* | KJ958482 | 1.33 | Bacillariophyta | *Unknown* |  | 1.02 | Unknown |
| 16 | *Ostreococcus sp.* | KF285533 | 0.80 | Chlorophyta | *Synechococcus sp.* | CT978603 | 1.12 | Cyanobacteria | *Phaeocystis globosa* | KC900889 | 0.99 | Haptophyta |
| 17 | *Dinophysis sp.* | KP826904 | 0.67 | Miozoa | *Unknown* |  | 1.07 | Unknown | *Synura sp.* | KM590725 | 0.91 | Ochrophyta |
| 18 | *Thalassiosira weissflogii* | KJ958485 | 0.64 | Bacillariophyta | *Chaetoceros simplex* | KJ958479 | 1.04 | Bacillariophyta | *Ostreococcus tauri* | KF285533 | 0.78 | Chlorophyta |
| 19 | *Dinophysis acuta* | KP826904 | 0.63 | Miozoa | *Synura sp.* | KM590725 | 0.91 | Ochrophyta | *Bathycoccus prasinos* | FO082259 | 0.75 | Chlorophyta |
| 20 | *Chrysochromulina sp.* | KJ201907 | 0.57 | Haptophyta | *Phaeocystis sp.* | KC900889 | 0.77 | Haptophyta | *Chaetoceros simplex* | KJ958479 | 0.71 | Bacillariophyta |
