# Supplementary material for: Genomic analysis of red-tide water bloomed with Heterosigma akashiwo in Geoje
Source: PeerJ. 2018 May 29;6:e4854. doi: 10.7717/peerj.4854 (PMC5983014; doi:10.7717/peerj.4854)
Supplement: Table S6 [file peerj-06-4854-s007.docx]

Supplementary 6. OTU richness, evenness, Shannon diversity index and Simpson diversity index of sampling sites based on OTU proportion

|  | Sites | Total OTU  (S) | Total proportion  (N) | OTU richness  (d) | Pielous evenness  (J’) | Shannon diversity index  (H’) | Simpson diversity index  (1-Lambda’) |
| --- | --- | --- | --- | --- | --- | --- | --- |
| 16S | Control | 61 | 100 | 13.03 | 0.6291 | 2.586 | 0.8254 |
|  | Edge | 103 | 100 | 22.15 | 0.6998 | 3.244 | 0.8863 |
|  | Bloom | 89 | 100 | 19.11 | 0.7095 | 3.185 | 0.8838 |
| 23S | Control | 67 | 100 | 14.33 | 0.6338 | 2.665 | 0.8505 |
|  | Edge | 82 | 100 | 17.59 | 0.6607 | 2.912 | 0.8437 |
|  | Bloom | 81 | 100 | 17.37 | 0.6594 | 2.898 | 0.8583 |
